# Extreme heat and cause-specific risk of hospital admission in the adult population in England: a case time series analysis

**DOI:** 10.1136/bmjopen-2025-105321

**Published:** 2026-06-23

**Authors:** Gillian Flower, Rebecca Cole, Arturo De La Cruz Libardi, Luis Mieiro, Jennifer K Quint, Antonio Gasparrini, Pierre Masselot

**Affiliations:** 1 Environment & Health Modelling (EHM) Lab, Department of Public Health, Environments and Society, London School of Hygiene & Tropical Medicine, London, UK; 2 Department of Health and Social Care, UK Government, London, UK; 3 Academic Centre for Healthy Ageing, Barts Health NHS Trust, London, UK; 4 School of Public Health, Imperial College London Faculty of Medicine, London, UK

**Keywords:** Hospitalization, Climate Change, EPIDEMIOLOGY, Health, PUBLIC HEALTH, Risk Factors

## Abstract

**Abstract:**

**Objectives:**

This study investigated the impact of heat on the risk of hospital admission due to a range of health conditions in England.

**Design:**

We used records of over 4 million hospital admissions in the summer months between 2008 and 2019, to construct daily time series of admissions in 32 837 census areas. Coupled with high-resolution environmental data, we conducted a case time-series analysis using distributed-lag non-linear models to measure the lagged relationship between summertime temperature and risk of admission for a broad set of health conditions. We derived the relative risks of admission at the 99th compared with the 50th temperature percentile to understand the effect of extreme heat in each locality.

**Setting:**

The adult population of England (aged 18 years and older).

**Outcome measures:**

Unplanned National Health Service (NHS) in-patient hospital admissions for cardiovascular, respiratory, genitourinary, metabolic and infectious diseases, along with their subcategories.

**Results:**

These conditions contributed more than 3.7 million admissions, of which over 1.5 million (42%) were for those aged 75 years and over. More than 80% of admissions were for respiratory, cardiovascular or genitourinary illness, which collectively contributed 3.1 million hospital admissions. There was clear evidence of an increased risk of hospital admission for many conditions, including acute renal failure (1.37, 95% CI 1.32 to 1.42), metabolic disorders (1.28, 95% CI 1.24 to 1.32), infectious and parasitic diseases (1.06, 95% CI 1.04 to 1.08), pneumonia (1.07, 95% CI 1.05 to 1.09) and chronic obstructive pulmonary disease (1.08, 95% CI 1.05 to 1.10). The evidence was less clear for asthma and diabetes, while there were negative associations for many cardiovascular conditions. There was a clear age gradient in heat-related admissions, with older people facing the greatest risk of admission.

**Conclusions:**

These findings highlight the widespread effect of extreme heat across a range of health conditions, in addition to mortality, and have implications for public health planning in our changing climate.

**STRENGTHS AND LIMITATIONS OF THIS STUDY**

- This study used a large database of administrative records from NHS Digital that included over 4 million hospital admissions from 2008 to 2019. This allowed a highly powered analysis of detailed diagnostic information, providing a comprehensive insight into hospitalisation events for the population of England, for the adult population.
- This study does not include emergency department attendances or admissions to psychiatric units, outpatient clinic visits, primary care utilisation or community-based care. Hospital admissions for planned or elective procedures were also excluded.
- We captured local differences in exposure to temperature and air pollution using a high-resolution spatial dataset.
- We used advanced statistical techniques to discriminate susceptibility to extreme heat while controlling for time-varying trends in admissions.

## Introduction

 Non-optimal temperature, and especially heat, is a known health hazard. Many studies have demonstrated that extreme heat increases mortality risk across a range of settings,^1–6^ but relatively few have considered the implications for healthcare and temperature-related hospital admission. This focus on mortality has created a gap in our understanding of the broader health implications of extreme heat, and an unknown heat-related healthcare demand. This potentially results in a substantive underestimation of the health burden of heat, in addition to high costs for the health system that would increase under the projected warming under climate change.

Globally, existing evidence suggests that temperature has a substantial impact on hospitalisation risk, and consequently incurs healthcare costs,^7 8^ although with considerable heterogeneity in vulnerability between and within populations, as well as across specific causes.^9–12^ There is also evidence of a distinct risk profile compared with temperature-related mortality.^13 14^ So far, evidence from the UK is more limited, with previous studies focusing on a small subset of conditions and/or populations.^15–18^

Over recent years, the UK has observed an unprecedented increase in the frequency of high temperatures, with the State of the UK Climate 2023 report warning that very hot days, of temperatures exceeding 30°C, have more than trebled since 1961 to 1990.^19^ Meanwhile, an ageing population with complex care needs means that demand for healthcare is increasing dramatically, putting the NHS under greater pressure than ever. Evidence from the King’s Fund also shows that the number of hospital beds has more than halved over the last 30 years.^20^ These factors place great importance on appropriate allocation of limited NHS resources. These societal and environmental changes mean it is critically important to understand individual vulnerabilities to extreme heat, to enable policy decisions that allow the provision of care to those with the greatest need. This comprehensive assessment for the UK is still lacking.

In this contribution, we examined the effect of heat during the summer months on the risk of admission for a broad range of health conditions. This work is enabled by detailed administrative records of admissions to NHS hospitals in England from 32 837 small-area census units in the period 2008–2019, linked with high-resolution daily temperature maps to capture local variation in exposure and vulnerability.

## Methods

### Hospital admissions data

We used administrative records from the NHS Hospital Episode Statistics (HES) Admitted Patient Care (APC) database, of more than 4 million unplanned hospital admissions that occurred in the summer months (June–August) between 2008 and 2019 across England. We used this information to construct time series of the daily number of hospital admissions for different age groups (18–64, 65–74, 75–84 and 85 and above) and health conditions (table 1), in each local area of England. We defined two levels of geographical aggregation using the UK Office for National Statistics (ONS) 2011 Census boundaries for 32 837 lower layer super output areas (LSOAs), each containing approximately 1600 residents, nested within 324 local authority districts (LADs).

**Table 1:** Hospital admissions by primary diagnosis the number of unplanned hospital admissions in the summer months (June to August) across England, by the primary diagnosis at admission

Diagnostic group (ICD-10 codes)	Total number of admissions	Diagnostic subgroup	ICD-10 codes	Number of admissions by subgroup
Respiratory (J00–99)	1 027 401	Acute respiratory infection	J00–06, J20–22	198 577
		Pneumonia	J12–18	356 263
		COPD	J40–44	218 296
		Asthma	J45–46	77 800
Cardiovascular (I00–99)	1 250 071	Stroke	I60–69	221 978
		Myocardial infarction	I21–23	171 006
		Heart failure	I50	138 515
		Hypotension	I95	49 211
Genitourinary (N00–99)	824 586	Renal disease	N00–30	643 284
		Acute renal failure	N17	88 697
Infectious and parasitic (A00–99, B00–99)	371 948	Bacterial diseases	A20–28	156 157
Endocrine, nutritional, metabolic (E00–99)	243 734	Metabolic disorders	E70–90	115 793
		Diabetes mellitus	E10–14	81 460

COPD, chronic obstructive pulmonary disease; ICD-10, International Classification of Diseases, 10th revision .

APC datasets capture visits to NHS hospital trusts in England that used a hospital bed, but do not include emergency department attendances (unless subsequently admitted), admissions to psychiatric units, outpatient visits, primary care utilisation or community-based care. We excluded planned or elective hospital admissions and admissions for patients residing outside England. Repeated hospital admissions, for patients admitted within 30 days of a prior admission, were also excluded. Demographic information was extracted to obtain patient residential, age and diagnostic information for each hospitalisation. For each hospital admission, we identified the health outcome using the primary diagnosis only, deemed the most representative of the admission, coded using the International Classification of Diseases, 10th revision (ICD-10). We selected the health conditions identified in the literature as having an etiological relationship with heat. To avoid misrepresentation of certain health conditions, we excluded hospital admissions associated with conditions more commonly reported via alternative NHS databases, such as the Emergency Care Dataset or the Mental Health Services Dataset. For this reason, we excluded mental health-related admissions and admissions due to wound or injury. The remaining diagnoses associated with the greatest number of admissions were prioritised for our analysis to ensure adequate power. See online supplemental appendix for a complete list of health conditions and their ICD codes, included in the analysis.

### Environmental data

We used the HadUK database from the Met Office to extract high-resolution daily temperature values over a 1×1 km grid. These gridded temperature values are reported to have a relatively small uncertainty range of 0.2 degrees.^21^ Daily measures of nitrogen dioxide (NO_2_) and fine particulate matter (PM_2.5_) over the same grid were reconstructed through a well-performing ensemble-based spatio-temporal machine learning model, with cross-validated R^2^ of 0.69 and 0.82, respectively.^22^ These data were used to calculate area-weighted daily mean temperature and pollution values for each LSOA in the study period.

### Statistical methods

We conducted a two-stage case time series analysis for each diagnosis, where LAD and age-specific models are fitted in the first stage, and the coefficients representing the exposure-response function of temperature are pooled in a second-stage meta-analysis model.^4^

More specifically, in the first stage, we fitted a quasi-Poisson conditional regression model for each LAD j and age group a, further stratified by LSOA i, and year/month k, represented by:


$$g\left[E\left(y_{jait}\right)\right]=\xi_{jaik}+f\left(x_{jit},l;\theta_{ja}\right)+s\left(t;\gamma_{ja}\right)+\sum_{p=1}^{P}g(z_{jit};\varphi_{ja})$$

Here, t is an index for individual days in each time series, g() is a logarithmic link function, and $\xi_{jait}$ is a strata-specific intercept that captures LSOA-specific seasonal and long-term trends.^23^ The function $f\left(x_{jit},l\right)$ specifies the exposure-lag-response relationship between daily LSOA-level temperature, $x_{jit}$, and each cause of admission, allowing for delayed effects, l, up to 3 days. The function is parametrised as a bidimensional cross-basis using a distributed lag non-linear model,^24^ with the exposure-response function defined through a natural spline with knots placed at the 50th and 90th percentiles of the LAD-specific temperature distribution, and the lag-response as a natural spline with 1 interior knot. We controlled for specific confounders $z_{jit}$, representing air pollution (PM_2.5_ and NO_2_), modelled linearly over lag 0–1, as well as day of the week as categorical indicators. Finally, we further controlled for temporal trends by directly modelling LAD-level seasonality with natural splines of day of the year with 4 df in $s\left(t;\gamma_{ja}\right)$.

In the second stage, the coefficients ${\hat{\theta }}_{ja}$, quantifying the temperature-response-lag relationship were reduced to create the coefficients ${\hat{\theta }}_{ja}$ quantifying the total effect cumulated over the lag period. These coefficients were then pooled using a random-effect multivariate meta-analysis to obtain the England-wide temperature-related relative risks (RRs) associated with each cause of admission. Results were primarily reported using effect summaries representing the RR at the 99th summer temperature percentile versus the 50th, chosen to illustrate the risk associated with extreme heat in each locality. The 50th temperature percentile was chosen as a reference point for RRs, instead of a minimum morbidity temperature, to aid comparability across health conditions.

In sensitivity analyses, we changed the definition of the cross-basis using a single knot at the 75th percentile of temperature for the exposure-response relationship, and then extended the lag period to 5 days (lag 0–5).

### Patient and public involvement

This study used routinely collected patient data from NHS Digital. Patients and the public were not involved in the design or conduct of this research.

## Results

### Hospital admission burden

**Figure 1:**
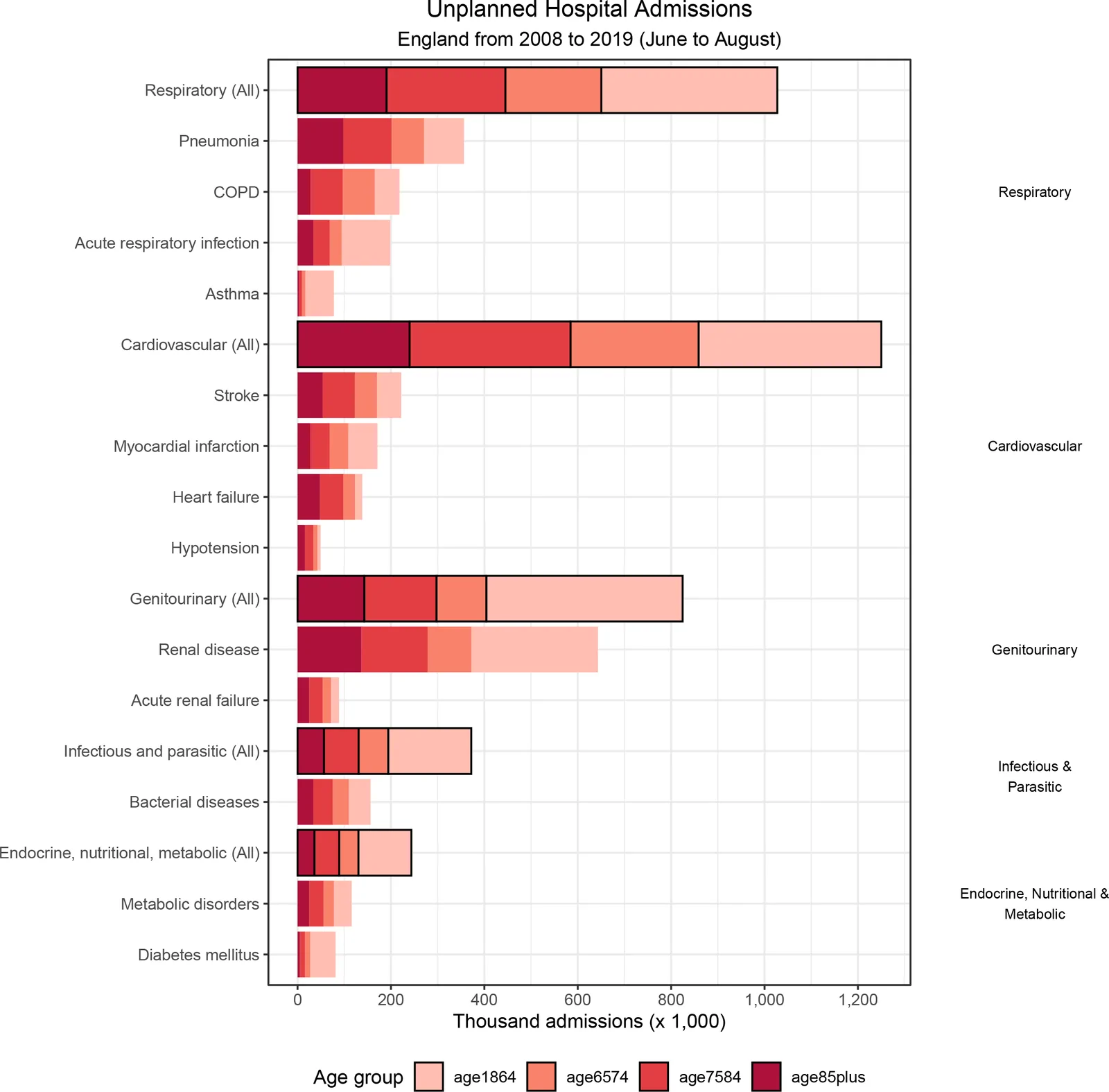
Hospital admission burden by age group. The total number of unplanned hospital admissions in the summer months (June to August) across England, by the primary diagnosis and age at admission. COPD, chronic obstructive pulmonary disease.

Table 1 reports the number of hospital admissions that occurred in England over the summer months of 2008–2019 by primary diagnosis. Figure 1 offers a graphical overview with further stratification by age group. Over this period, the selected causes of hospitalisation contributed more than 3.7 million admissions, of which over 1.5 million (42%) were for those aged 75 years and over. More than 80% of admissions were for respiratory, cardiovascular or genitourinary illness, which collectively contributed 3.1 million hospital admissions, 1.5 million (48%) of which were for those aged 75 years and over. Infectious and parasitic illnesses, as well as endocrine, nutritional and metabolic conditions, also contributed a substantial number of admissions over the study period, with 0.3 million and 0.2 million admissions, respectively. Of the 1.0 million admissions for respiratory conditions, 0.8 million were for acute respiratory infection (ARI), pneumonia or chronic obstructive pulmonary disease (COPD), with these conditions each contributing 0.2 million, 0.4 million and 0.3 million admissions, respectively. Stroke and myocardial infarction (MI) were the main contributors to cardiovascular-related hospital admissions, each contributing 0.2 million hospital admissions. Genitourinary illness was responsible for 0.8 million admissions over the study period, 0.6 million of which were for renal disease.

### Temperature-related risk of admission

**Figure 2:**
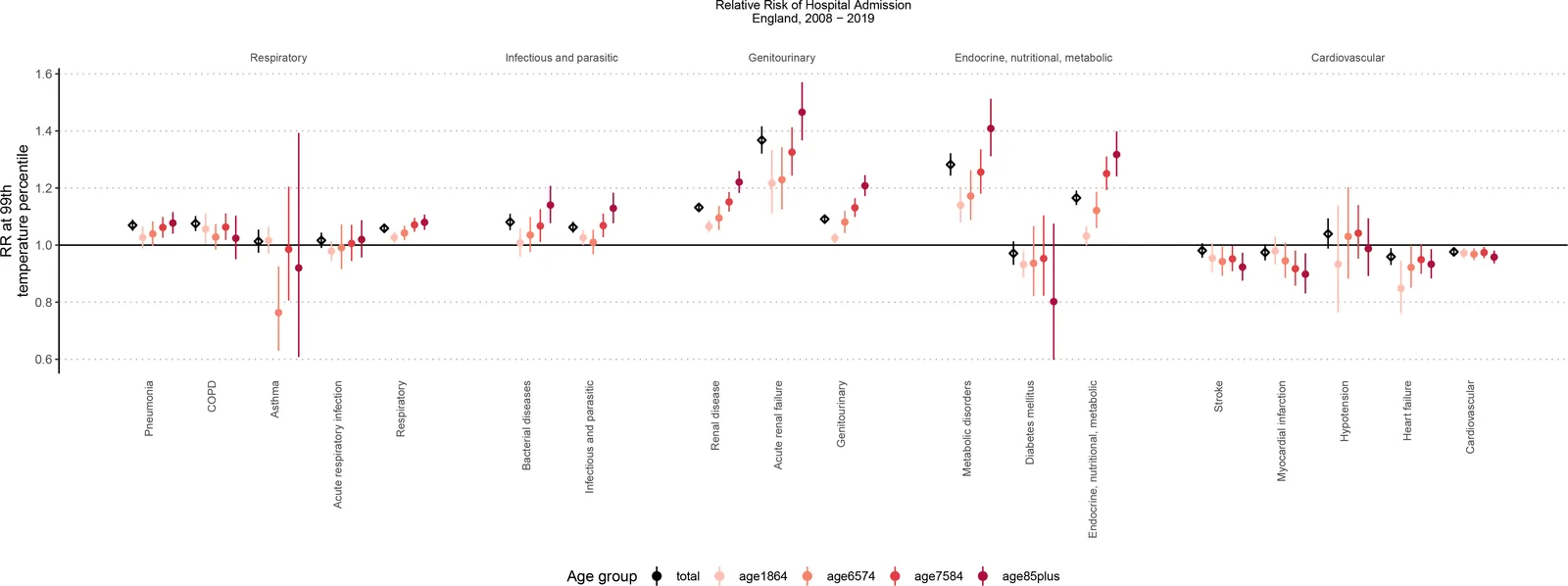
Relative risk (RR) of hospital admission risk of hospital admission at the 99th temperature percentile relative to the 50th percentile, by age group and cause of admission. COPD, chronic obstructive pulmonary disease.

Figure 2 shows the RR of hospital admission (99th vs 50th temperature percentile) for each cause of admission and age group. The 50th temperature percentile was chosen as a reference point to aid comparability of RRs for different health conditions. The full exposure-response relationships are reported in online supplemental figures S1–S19. The graph indicates clear evidence of associations between extreme heat and risk of hospital admission for some causes, namely acute renal failure, metabolic disorders, infectious and parasitic diseases, pneumonia and COPD. However, the evidence is less clear-cut for other causes, for instance asthma and diabetes, while the analysis reported consistent negative associations with many cardiovascular causes. The comparison of the estimates across age groups indicates a clear increase in risk with age, with people aged 85 and older having a substantially higher RR of heat-related admission for many causes.

There was strong evidence of an increased risk for genitourinary illness (1.09, 95% CI 1.08 to 1.11), with risks increasing with age. For acute renal failure, there was a substantial increase in risk of admission across all age groups (1.37, 95% CI 1.32 to 1.42). There was strong evidence of an increased risk for endocrine, nutritional and metabolic-related hospital admission (1.17, 95% CI 1.14 to 1.19), with the highest risk for those aged 85 and older (1.32, 95% CI 1.24 to 1.40). There was an increased risk of infectious and parasitic-related admission for those aged 0 to 64 and 75 years and older, with the greatest risk among those aged 85 and older (1.13, 95% CI 1.08 to 1.18).

While there was no evidence of a temperature effect for ARI and asthma, there was clear evidence of an increased risk of COPD (1.08, 95% CI 1.05 to 1.10) and pneumonia (1.07, 95% CI 1.05 to 1.09) related admission. Estimates for diabetes-related hospitalisation risk had considerable uncertainty, with no evidence of an increased or decreased risk at the overall level. Lower RRs were observed in the youngest age group (0.93, 95% CI 0.89 to 0.98).

Results show a lower risk of hospital admission for cardiovascular illness under extreme heat. For MI and stroke, our study does not provide clear evidence of an increased or decreased risk under extreme heat at the overall level (0.97, 95% CI 0.97 to 1.00, and 0.98, 95% CI 0.96 to 1.01, respectively), but lower RRs were observed in the oldest age groups. The sensitivity analysis showed largely consistent results when modifying the definition of the exposure-response function and the lag period (see online supplemental table S1).

## Discussion

This study provides an extensive assessment of heat-related hospital admission risk across a broad range of conditions for residents of England in the period 2008–2019, using an extensive database and advanced statistical methods. Our findings show that extreme heat increases the risk of admission for a range of conditions, with clear evidence for endocrine, nutritional and metabolic illnesses, genitourinary conditions including acute renal failure, and some respiratory conditions such as COPD and pneumonia. Surprisingly, results indicate a lower risk of hospital admission for many cardiovascular outcomes. We found an age gradient in hospital admission risks for many conditions, with the oldest age groups showing the highest risk of admission during extreme heat.

Although there are relatively few UK-based studies reporting cause-specific temperature-related hospitalisation risk, the findings of this study are broadly consistent with existing evidence. Rizmie *et al* also found clear evidence of an increased risk for metabolic, infectious and respiratory diseases at temperatures of 25°C and above, with a similar increase in age-related risk.^25^ Konstantinoudis *et al* assessed the risk of COPD-related hospitalisation and found a 1.47% increase in hospitalisation risk for every 1 degree above 23.2°C.^16^ while Konstantinoudis *et al* similarly found inconclusive evidence of asthma-related hospital admission for the period 2008 to 2013, despite evidence of a 2.96% increase in admission risk for every 1 degree increase in ambient temperature in the period 2002 to 2007.^15^ In Spain, Achebak *et al* found an increased risk for infectious and parasitic illness, endocrine and metabolic conditions, renal failure and some respiratory conditions, including COPD and pneumonia.^10^ Zhao *et al* compared demographic differences in hospitalisation risk in Brazil and found that infectious and parasitic disease, and endocrine, nutritional and metabolic conditions saw the greatest percentage increase in hospitalisation under heat exposure.^26^ In a study of US Medicare beneficiaries aged 65 and above, Gronlund *et al* showed that extreme heat was linked to a 15% and 4% increase in admissions for renal and respiratory diseases, respectively.^27^

Interestingly, other studies obtained similar negative associations for cardiovascular-related admissions, with estimates consistent with our analysis.^10 13 25–27^ A possible explanation is prehospitalisation mortality, either in the community or during emergency department attendance. We would expect preadmission mortality to decrease the perceived risk of hospitalisation, particularly for acute events such as MI or stroke, and among older patients where mortality risk is greater, both of which were observed in this study. Kovats *et al* noted that we would expect total heat-related admissions to at least match heat-related mortality in magnitude, yet this was not found in their study, despite evidence of increased admissions for respiratory and renal disease.^28^ It is likely that this shortfall in admissions at the overall level is a result of pre-hospital mortality for other conditions such as cardiovascular disease. It is worth noting that patients experiencing suspected MI or stroke are prioritised for ambulance response, so it is plausible that a higher proportion of these patients are managed via emergency department care. Some of these patients may also be managed via dedicated outpatient clinical pathways designed to prevent hospital admission. Further work, taking a system-wide approach across multiple sources of health outcome data, is necessary in order to fully distinguish these patterns of cardiovascular hospitalisation.

While this study provides substantial insight into hospitalisation risk across England, there are a few limitations to acknowledge. Due to the complex nature of healthcare systems both in the UK and globally, findings from this study are not directly comparable to populations in other countries, which may present with very different exposure patterns, sociodemographic characteristics, and thresholds for hospital admission. Although this study benefits from high resolution mapping of temperature exposure, there remains a minor risk of exposure misclassification, especially in the more rural LSOAs. More importantly, this study reports country-wide estimates and did not attempt to identify local differences in risks within England. Additionally, while APC tables provide extensive information regarding healthcare usage across England, they do not provide a complete picture of health needs for the population. Furthermore, key omissions include interactions with mental health services, out-patient clinic visits, community-based care or prehospital mortality. Importantly, this work does not consider paediatric heat related risk of admission. Further work is needed to understand these as-yet unseen demands, as well as economic costs, to recognise the full impact of extreme heat on the population of England. Finally, note that some relevant covariates could not be included for lack of data availability, such as ozone which might interact with temperature and impact respiratory diseases, among others.

Our findings have important implications for managing conditions and vulnerable groups at greater risk from extreme heat. They also underscore the urgent need for targeted public health interventions to protect the health of older adults, such as implementing risk-based strategies for vulnerable individuals, enhancing community outreach, and ensuring hospital preparedness to mitigate the adverse health impacts of extreme heat. While high temperatures have known implications for some health conditions, this study quantifies those impacts as hospitalisation risk. In considering a broad range of diagnoses, we are also able to provide insight regarding admission risk for conditions where the relationship with extreme heat is less clear or may have been overlooked. In both situations, it is important that all of the results from this study are interpreted alongside a knowledge of the healthcare system from which they originate, and the administrative procedures governing HES databases.

## Supplementary material

10.1136/bmjopen-2025-105321 online supplemental file 1

## Data Availability

Data may be obtained from a third party and are not publicly available.
