# Supplementary material for: Extreme heat and cause-specific risk of hospital admission in the adult population in England: a case time series analysis
Source: BMJ Open. 2026 Jun 23;16(6):e105321. doi: 10.1136/bmjopen-2025-105321 (PMC13295835; doi:10.1136/bmjopen-2025-105321)
Supplement: online supplemental file 1 [file bmjopen-16-6-s001.pdf]

Supplementary Material

Extreme heat and cause-specific risk of hospital admission in the adult population in England: a case time series analysis

Gillian Flower*[1,2], Rebecca Cole[1], Luis Mieiro[1,4], Jennifer K Quint[3], Antonio Gasparrini[1] and Pierre Masselot[1]

[1] Environment & Health Modelling (EHM) Lab, Department of Public Health, Environments and Society, London School of Hygiene & Tropical Medicine, London, United Kingdom

[2] Department of Health and Social Care, UK Government

[3] School of Public Health, Faculty of Medicine, Imperial College London

[4] Academic Centre for Healthy Ageing, Barts Health NHS Trust, London, United Kingdom

Contents:

(A) Supplementary figures (S1-S18) showing the estimated temperature-response curves from the main analysis – the risk of in-patient hospital admission by cause of admission and age group

(B) Table S1 showing results of sensitivity analysis conducted based on two scenarios, alongside results of the main analysis:

  I. Adjustment to the exposure-response function to use 1 knot placed at the 75th percentile

  II. Extension of the lag-period to 5 days (with 1 internal knot)

(A) Supplementary Figures S1-S18

Estimated exposure-response curves describing the relationship between temperature and risk of in-patient hospital admission, by cause of admission and age group

Please note that the 'total' in the subsequent plots refers to the adult population only.

Fig. S1 | Respiratory (all)

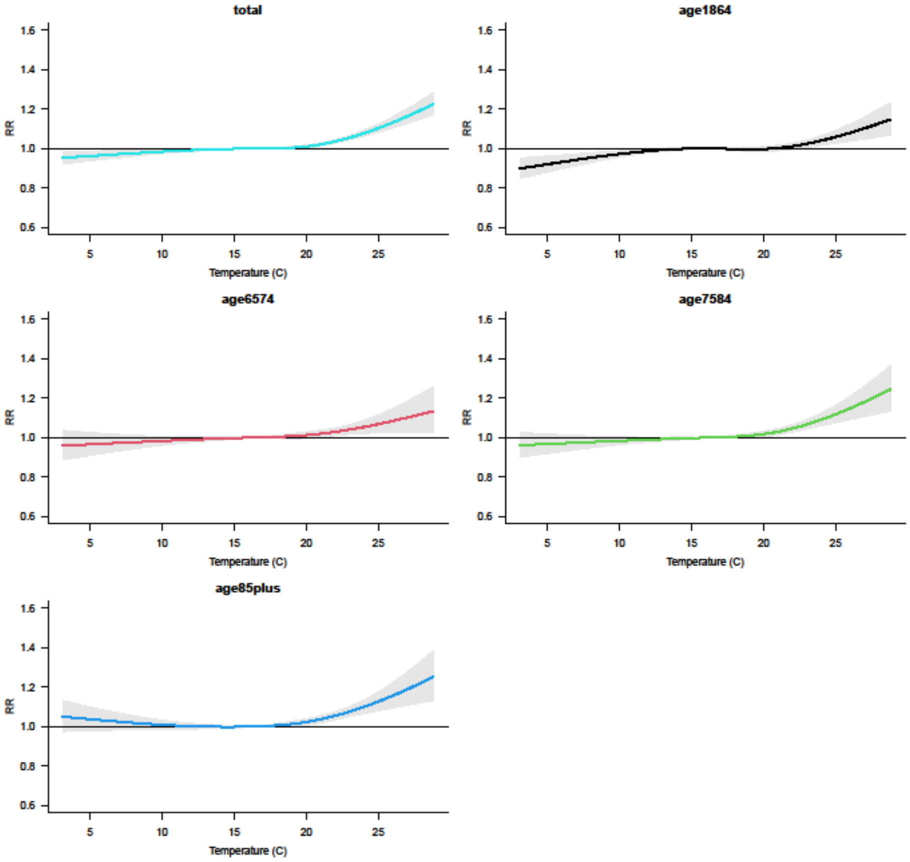

Fig. S2 | Acute respiratory infection

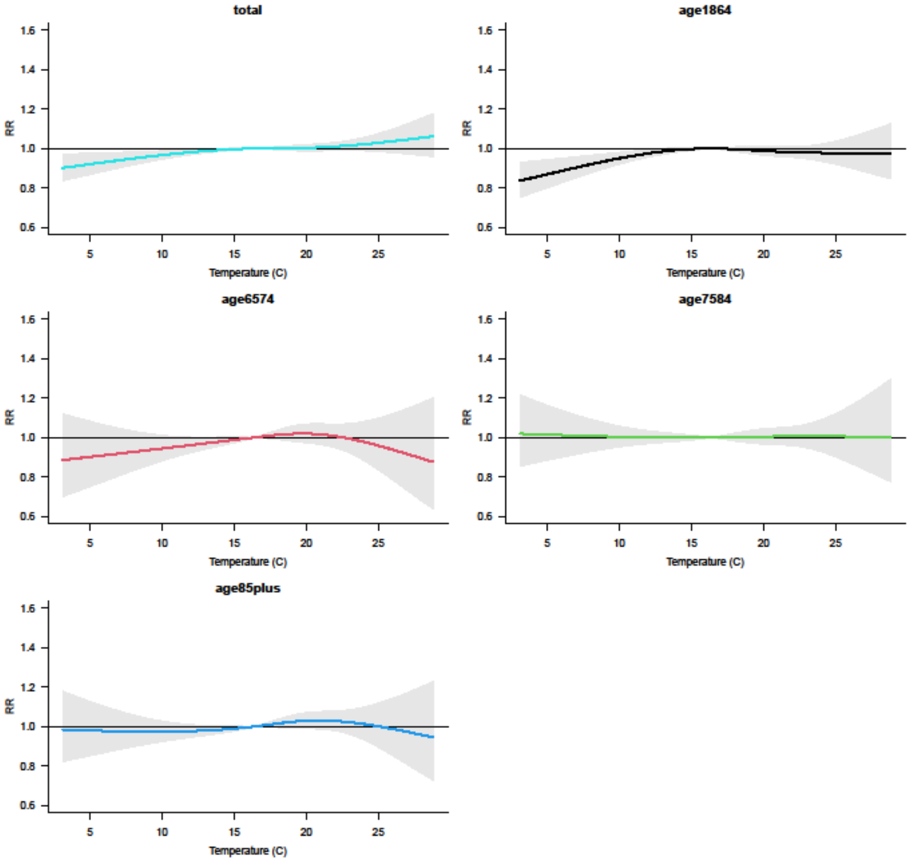

Fig. S3 | Pneumonia

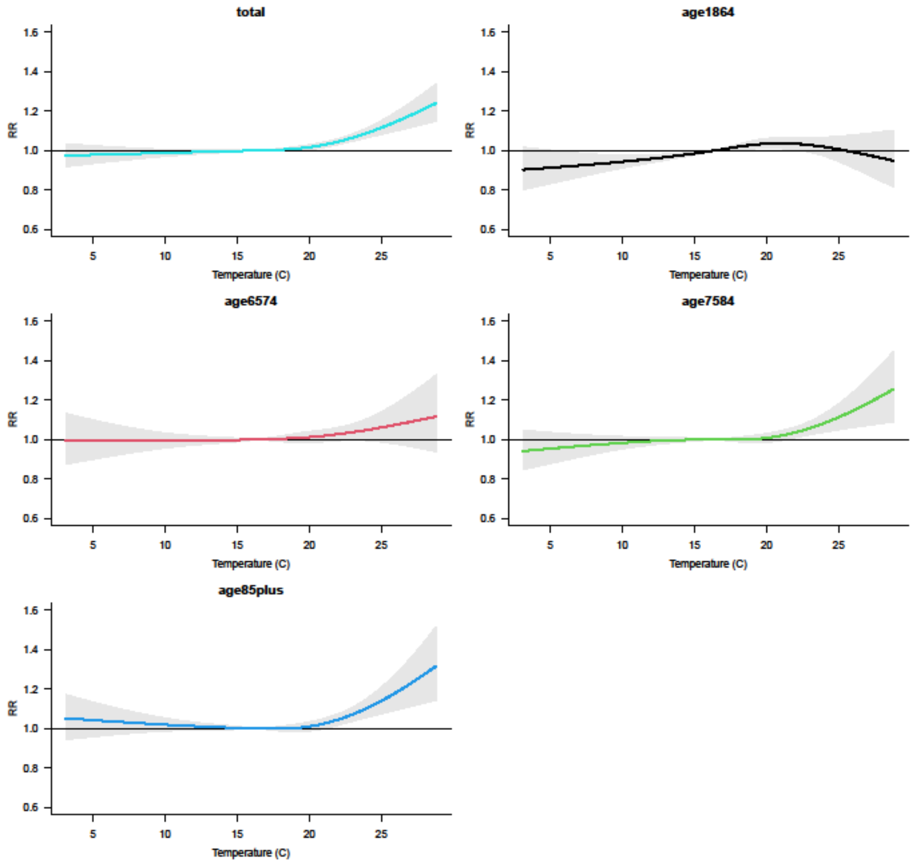

Fig. S4 | Chronic obstructive pulmonary disease

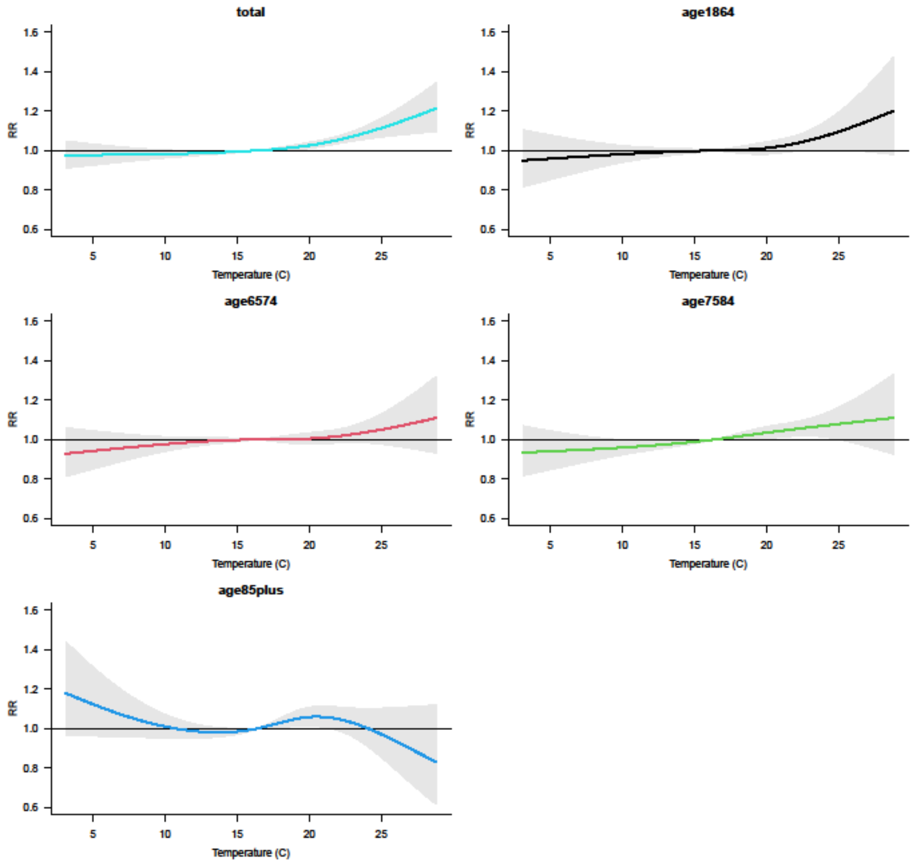

Fig. S5 | Asthma

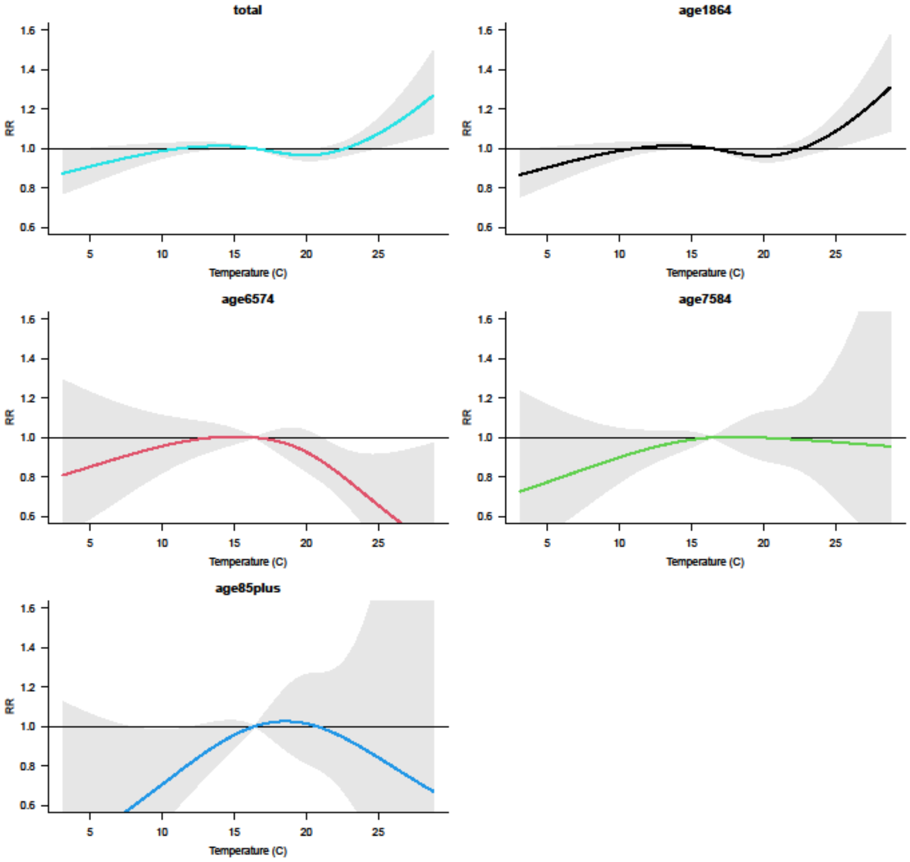

Fig. S6 | Cardiovascular (all)

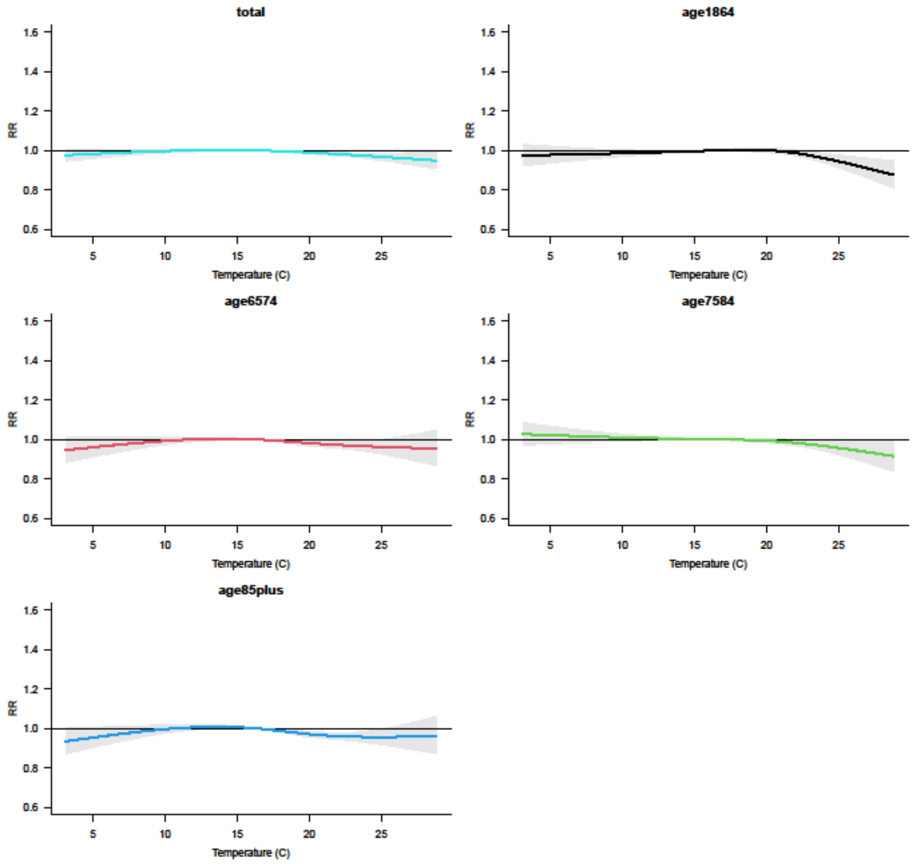

Fig. S7 | Stroke

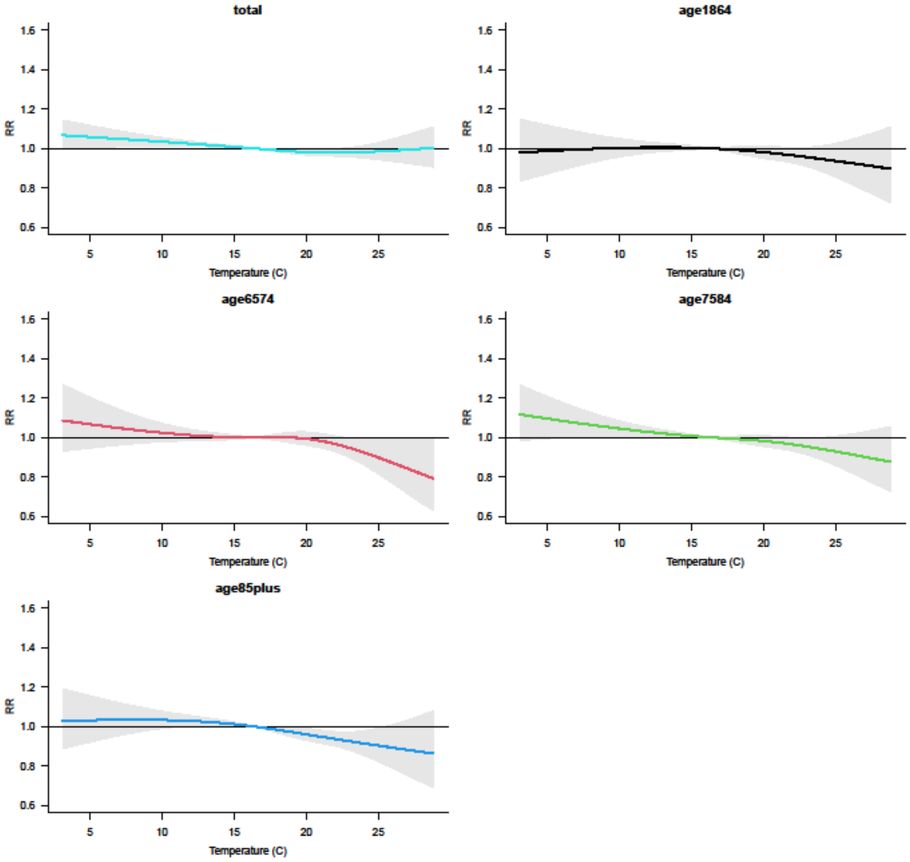

Fig. S8 | Myocardial infarction

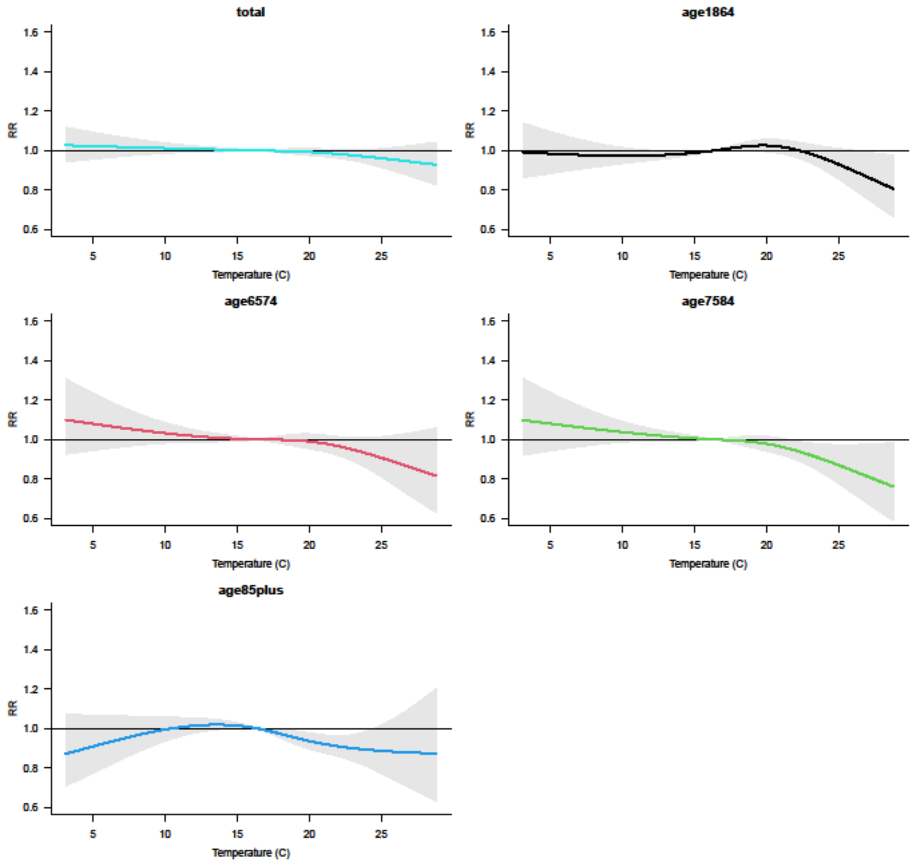

Fig. S9 | Heart failure

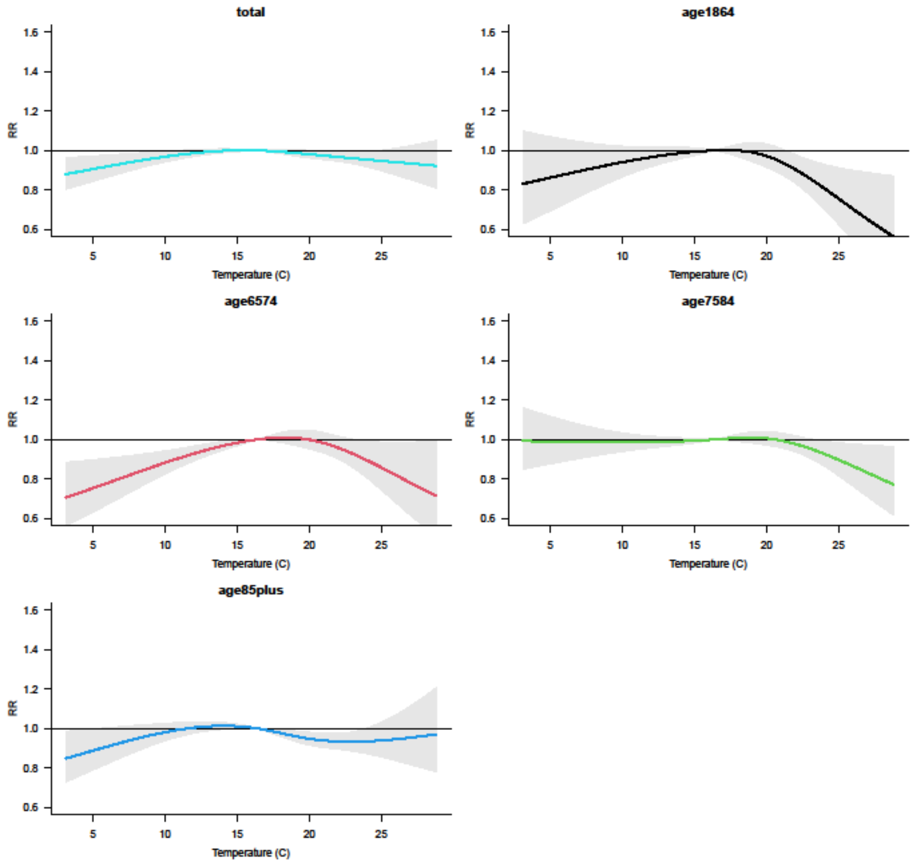

Fig. S10 | Hypotension

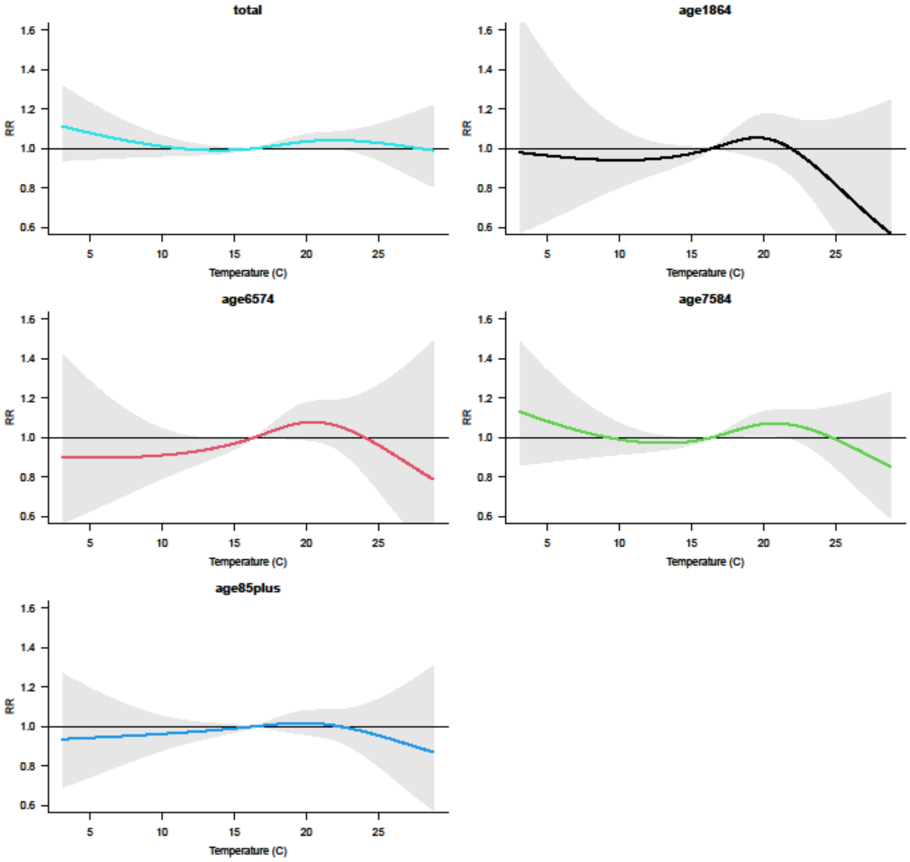

Fig. S11 | Genitourinary (all)

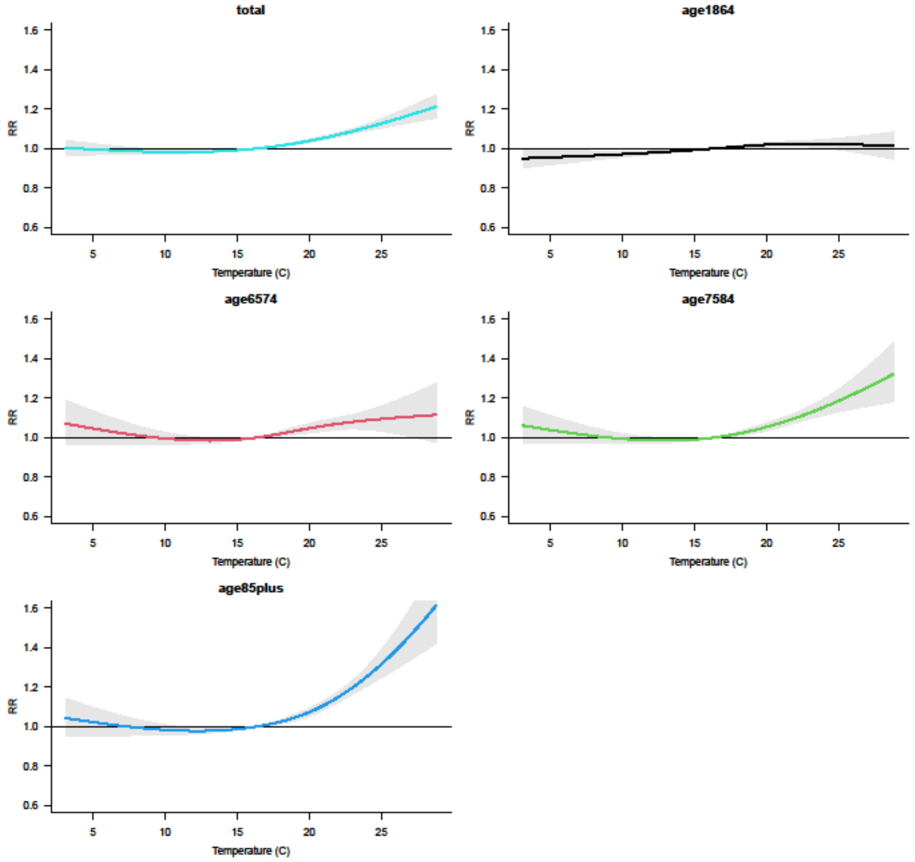

Fig. S12 | Renal disease

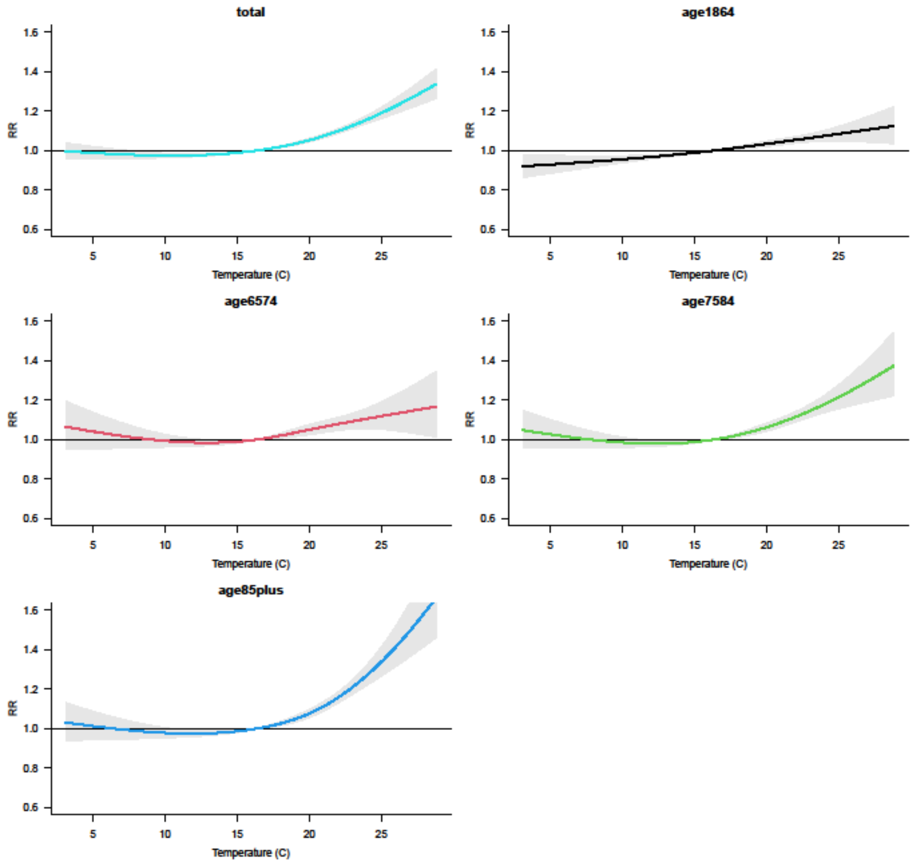

Fig. S13 | Acute renal failure

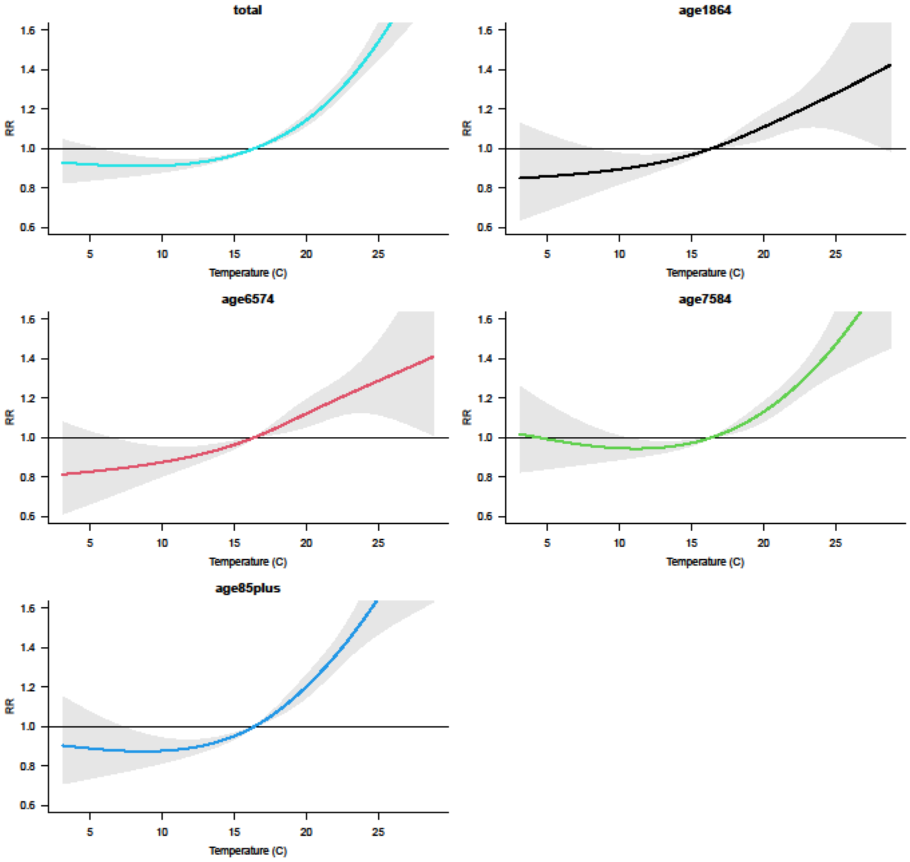

Fig. S14 | Infectious and parasitic (all)

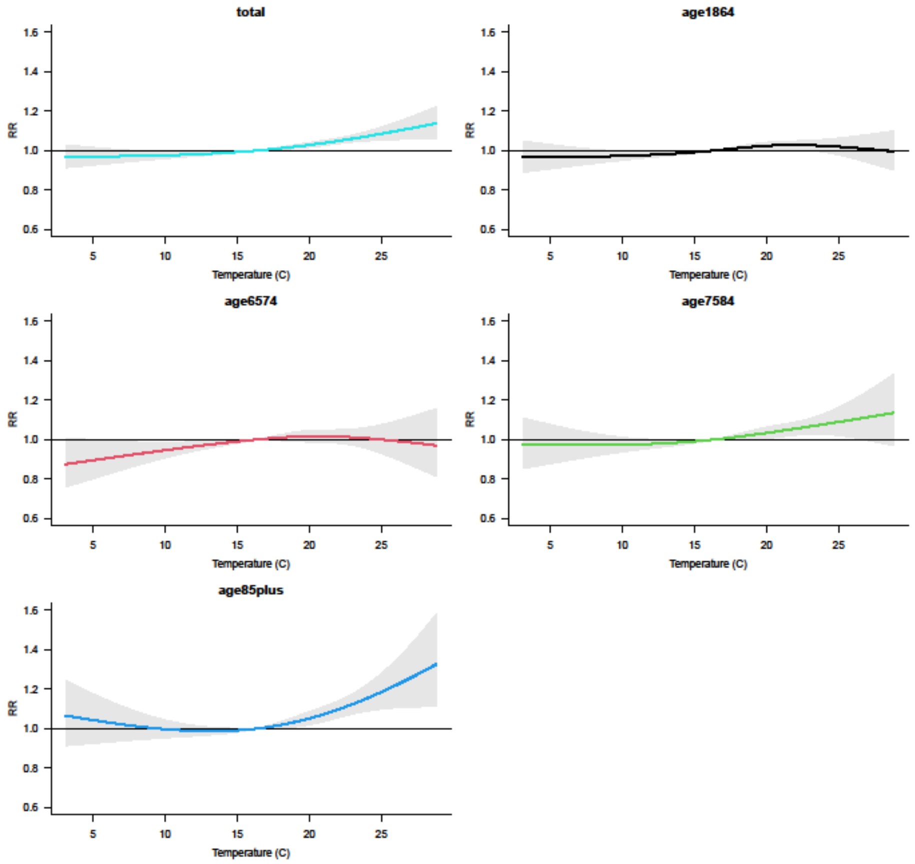

Fig. S15 | Bacterial diseases

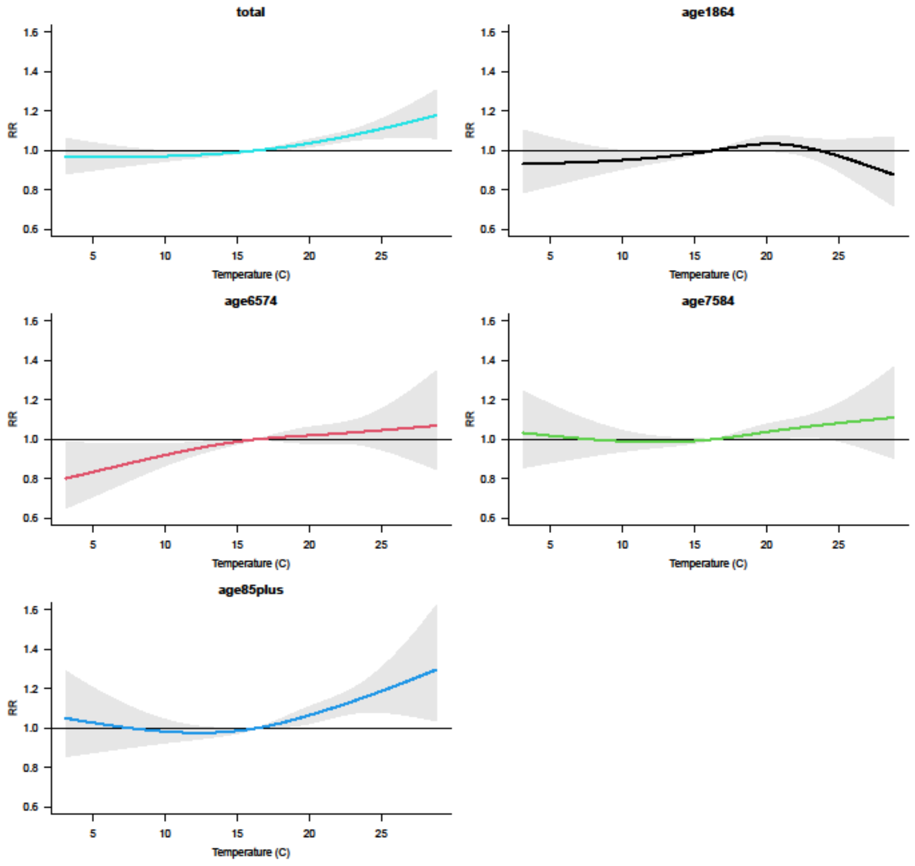

Fig. S16 | Endocrine, nutritional and metabolic (all)

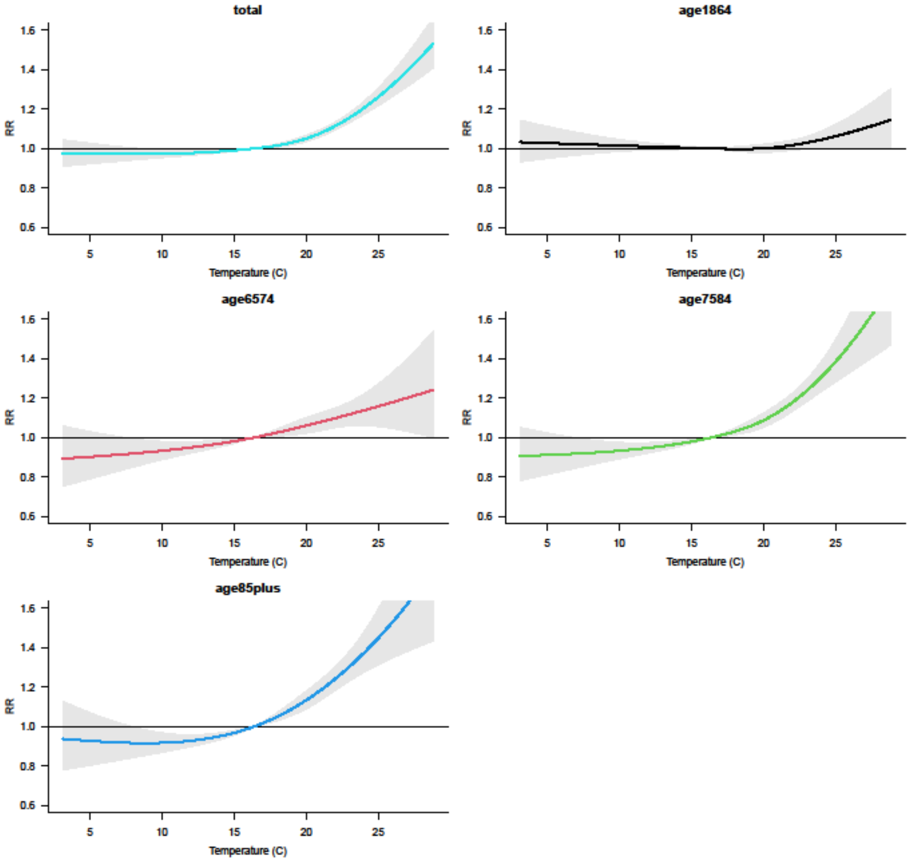

Fig. S17 | Metabolic disorders

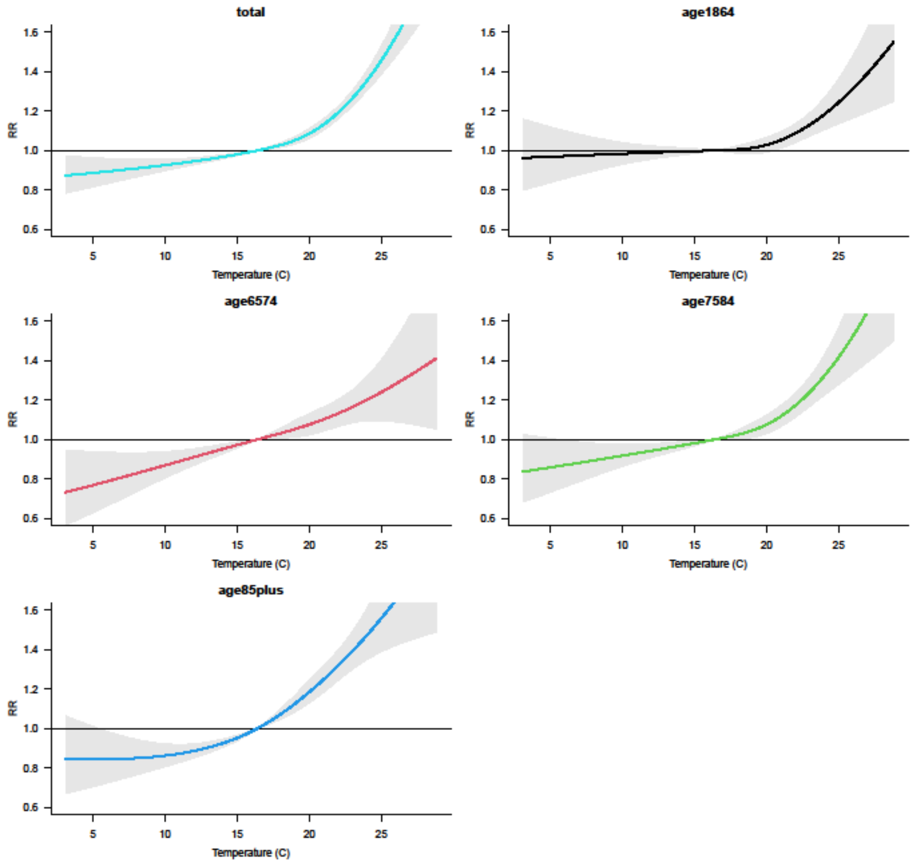

Fig. S18 | Diabetes mellitus

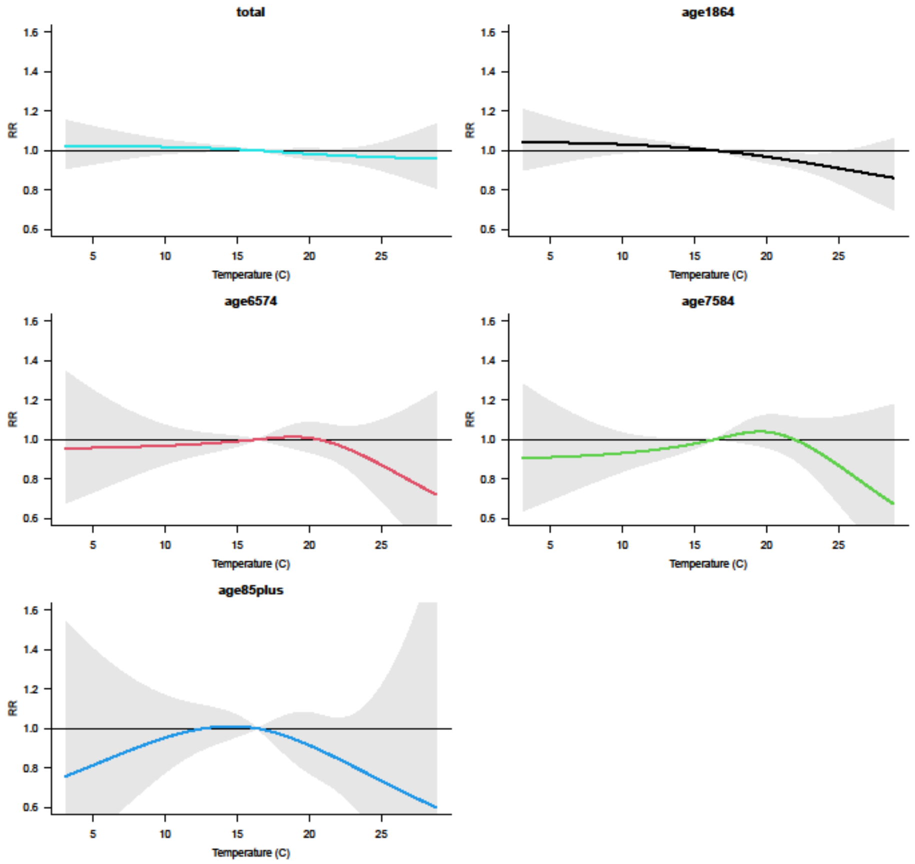

(B) Table S1 Sensitivity Analysis

Table S1 provides results of sensitivity analysis conducted based on two alternative model parameterisations, alongside results of the main analysis:

I. Adjustment to the exposure-response function to use 1 knot placed at the 75th percentile

II. Extension of the lag-period to 5 days (with 1 internal knot)

Diagnostic Group	Diagnostic Sub-Group	Age Group	Main analysis	I. Sensitivity (ERF)	II. Sensitivity (lag)
Cardiovascular (I00-99)	Cardiovascular (I00-99)	Age 18-64	0.972, CI: 0.953 - 0.991	0.976, CI: 0.956 - 0.996	0.977, CI: 0.956 - 0.999
		Age 65-74	0.968, CI: 0.947 - 0.989	0.971, CI: 0.95 - 0.994	0.971, CI: 0.947 - 0.996
		Age 75-84	0.974, CI: 0.955 - 0.994	0.981, CI: 0.961 - 1.001	0.983, CI: 0.96 - 1.006
		Age 85+	0.958, CI: 0.936 - 0.981	0.96, CI: 0.937 - 0.984	0.948, CI: 0.922 - 0.974
		All adults	0.976, CI: 0.966 - 0.987	0.977, CI: 0.966 - 0.987	0.981, CI: 0.969 - 0.992
	Heart failure (I50)	Age 18-64	0.849, CI: 0.761 - 0.946	0.845, CI: 0.756 - 0.943	0.781, CI: 0.685 - 0.89
		Age 65-74	0.922, CI: 0.85 - 0.999	0.932, CI: 0.86 - 1.011	0.886, CI: 0.808 - 0.971
		Age 75-84	0.949, CI: 0.9 - 1.001	0.964, CI: 0.913 - 1.017	0.914, CI: 0.857 - 0.974
		Age 85+	0.933, CI: 0.883 - 0.986	0.947, CI: 0.895 - 1.001	0.891, CI: 0.833 - 0.953
		All adults	0.959, CI: 0.93 - 0.989	0.963, CI: 0.932 - 0.994	0.929, CI: 0.894 - 0.966
	Hypotension (I95)	Age 18-64	0.933, CI: 0.764 - 1.139	0.913, CI: 0.751 - 1.11	0.856, CI: 0.679 - 1.081
		Age 65-74	1.03, CI: 0.882 - 1.203	1.022, CI: 0.88 - 1.188	0.994, CI: 0.826 - 1.196
		Age 75-84	1.042, CI: 0.952 - 1.14	1.061, CI: 0.97 - 1.16	1.009, CI: 0.905 - 1.125
		Age 85+	0.988, CI: 0.892 - 1.094	1.009, CI: 0.913 - 1.116	0.977, CI: 0.869 - 1.098
		All adults	1.039, CI: 0.988 - 1.094	1.068, CI: 1.014 - 1.125	1.039, CI: 0.976 - 1.106
	Myocardial infarction (I21-23)	Age 18-64	0.98, CI: 0.932 - 1.029	1.001, CI: 0.952 - 1.053	0.968, CI: 0.913 - 1.027
		Age 65-74	0.945, CI: 0.885 - 1.009	0.968, CI: 0.905 - 1.035	0.929, CI: 0.859 - 1.004
		Age 75-84	0.917, CI: 0.858 - 0.981	0.931, CI: 0.869 - 0.997	0.935, CI: 0.867 - 1.009
		Age 85+	0.898, CI: 0.831 - 0.971	0.897, CI: 0.829 - 0.97	0.852, CI: 0.773 - 0.94
		All adults	0.974, CI: 0.946 - 1.003	0.985, CI: 0.956 - 1.015	0.979, CI: 0.946 - 1.013
	Stroke (I60-69)	Age 18-64	0.954, CI: 0.904 - 1.007	0.983, CI: 0.929 - 1.039	0.969, CI: 0.912 - 1.031
		Age 65-74	0.943, CI: 0.892 - 0.995	0.966, CI: 0.915 - 1.021	0.943, CI: 0.884 - 1.006
		Age 75-84	0.952, CI: 0.908 - 0.997	0.974, CI: 0.928 - 1.022	0.962, CI: 0.912 - 1.015
		Age 85+	0.923, CI: 0.875 - 0.973	0.942, CI: 0.892 - 0.994	0.914, CI: 0.86 - 0.972
		All adults	0.981, CI: 0.956 - 1.006	0.99, CI: 0.964 - 1.017	0.994, CI: 0.964 - 1.024
Endocrine, nutritional, metabolic (E00-99)	Diabetes mellitus (E10-14)	Age 18-64	0.932, CI: 0.886 - 0.98	0.944, CI: 0.896 - 0.995	0.927, CI: 0.875 - 0.982
		Age 65-74	0.936, CI: 0.821 - 1.067	0.96, CI: 0.849 - 1.086	0.835, CI: 0.706 - 0.989
		Age 75-84	0.953, CI: 0.822 - 1.104	0.973, CI: 0.846 - 1.119	0.907, CI: 0.767 - 1.071
		Age 85+	0.802, CI: 0.598 - 1.075	0.806, CI: 0.618 - 1.05	0.756, CI: 0.547 - 1.044
		All adults	0.971, CI: 0.931 - 1.013	0.982, CI: 0.94 - 1.026	0.97, CI: 0.925 - 1.018
	Endocrine, nutritional, metabolic (E00-99)	Age 18-64	1.032, CI: 0.999 - 1.066	1.047, CI: 1.012 - 1.082	1.033, CI: 0.995 - 1.073
		Age 65-74	1.121, CI: 1.059 - 1.187	1.149, CI: 1.083 - 1.218	1.135, CI: 1.059 - 1.217
		Age 75-84	1.25, CI: 1.193 - 1.311	1.277, CI: 1.217 - 1.341	1.295, CI: 1.225 - 1.369
		Age 85+	1.317, CI: 1.24 - 1.399	1.346, CI: 1.265 - 1.432	1.381, CI: 1.288 - 1.481
		All adults	1.165, CI: 1.14 - 1.191	1.179, CI: 1.153 - 1.206	1.199, CI: 1.168 - 1.23
	Metabolic disorders (E70-90)	Age 18-64	1.14, CI: 1.08 - 1.203	1.178, CI: 1.114 - 1.246	1.173, CI: 1.098 - 1.252
		Age 65-74	1.172, CI: 1.088 - 1.263	1.208, CI: 1.119 - 1.303	1.186, CI: 1.084 - 1.298
		Age 75-84	1.256, CI: 1.18 - 1.336	1.295, CI: 1.217 - 1.379	1.285, CI: 1.193 - 1.383
		Age 85+	1.409, CI: 1.312 - 1.513	1.433, CI: 1.331 - 1.542	1.469, CI: 1.349 - 1.599
		All adults	1.282, CI: 1.243 - 1.322	1.308, CI: 1.269 - 1.349	1.346, CI: 1.297 - 1.396
Genitourinary (N00-99)	Acute renal failure (N17)	Age 18-64	1.217, CI: 1.11 - 1.333	1.228, CI: 1.118 - 1.349	1.204, CI: 1.075 - 1.348
		Age 65-74	1.229, CI: 1.125 - 1.343	1.257, CI: 1.152 - 1.371	1.282, CI: 1.156 - 1.421
		Age 75-84	1.325, CI: 1.243 - 1.413	1.355, CI: 1.268 - 1.447	1.351, CI: 1.248 - 1.462
		Age 85+	1.466, CI: 1.367 - 1.571	1.498, CI: 1.397 - 1.606	1.469, CI: 1.351 - 1.597
		All adults	1.368, CI: 1.321 - 1.416	1.391, CI: 1.341 - 1.443	1.417, CI: 1.357 - 1.481

	Genitourinary (N00-99)	Age 18-64	1.024, CI: 1.007 - 1.042	1.026, CI: 1.008 - 1.044	1.039, CI: 1.019 - 1.059
		Age 65-74	1.081, CI: 1.043 - 1.12	1.096, CI: 1.056 - 1.137	1.106, CI: 1.062 - 1.152
		Age 75-84	1.131, CI: 1.099 - 1.164	1.145, CI: 1.111 - 1.181	1.138, CI: 1.1 - 1.178
		Age 85+	1.208, CI: 1.172 - 1.245	1.231, CI: 1.193 - 1.271	1.212, CI: 1.17 - 1.256
		All adults	1.091, CI: 1.078 - 1.105	1.096, CI: 1.082 - 1.11	1.107, CI: 1.092 - 1.123
	Renal disease (N00-30)	Age 18-64	1.066, CI: 1.044 - 1.088	1.069, CI: 1.046 - 1.092	1.083, CI: 1.056 - 1.11
		Age 65-74	1.095, CI: 1.054 - 1.138	1.111, CI: 1.067 - 1.157	1.114, CI: 1.065 - 1.165
		Age 75-84	1.151, CI: 1.118 - 1.186	1.167, CI: 1.131 - 1.204	1.158, CI: 1.117 - 1.199
		Age 85+	1.221, CI: 1.183 - 1.26	1.245, CI: 1.205 - 1.287	1.22, CI: 1.176 - 1.266
		All adults	1.132, CI: 1.116 - 1.148	1.139, CI: 1.123 - 1.156	1.147, CI: 1.128 - 1.167
Infectious and parasitic (A00-99, B00-99)	Bacterial diseases (A20-28)	Age 18-64	1.007, CI: 0.959 - 1.058	1.016, CI: 0.965 - 1.069	0.995, CI: 0.939 - 1.054
		Age 65-74	1.035, CI: 0.976 - 1.099	1.058, CI: 0.995 - 1.124	0.994, CI: 0.926 - 1.067
		Age 75-84	1.067, CI: 1.011 - 1.126	1.073, CI: 1.014 - 1.136	1.042, CI: 0.975 - 1.114
		Age 85+	1.14, CI: 1.076 - 1.208	1.166, CI: 1.099 - 1.238	1.139, CI: 1.064 - 1.221
		All adults	1.081, CI: 1.052 - 1.11	1.091, CI: 1.061 - 1.122	1.077, CI: 1.043 - 1.113
	Infectious and parasitic (A00-99, B00-99)	Age 18-64	1.026, CI: 1 - 1.052	1.032, CI: 1.005 - 1.06	1.036, CI: 1.005 - 1.069
		Age 65-74	1.01, CI: 0.968 - 1.054	1.023, CI: 0.979 - 1.07	0.986, CI: 0.935 - 1.04
		Age 75-84	1.068, CI: 1.028 - 1.11	1.077, CI: 1.034 - 1.122	1.07, CI: 1.021 - 1.122
		Age 85+	1.129, CI: 1.077 - 1.184	1.154, CI: 1.099 - 1.213	1.13, CI: 1.07 - 1.193
		All adults	1.062, CI: 1.043 - 1.082	1.069, CI: 1.049 - 1.089	1.071, CI: 1.048 - 1.095
Respiratory (J00-99)	Acute respiratory infection (J00-06, J20-22)	Age 18-64	0.979, CI: 0.945 - 1.014	0.979, CI: 0.944 - 1.016	0.958, CI: 0.92 - 0.998
		Age 65-74	0.991, CI: 0.915 - 1.072	0.997, CI: 0.92 - 1.081	0.983, CI: 0.897 - 1.077
		Age 75-84	1.006, CI: 0.944 - 1.071	1.022, CI: 0.958 - 1.09	0.953, CI: 0.884 - 1.027
		Age 85+	1.02, CI: 0.957 - 1.087	1.035, CI: 0.969 - 1.105	0.98, CI: 0.905 - 1.061
		All adults	1.016, CI: 0.99 - 1.044	1.016, CI: 0.988 - 1.045	1.004, CI: 0.972 - 1.037
	Asthma (J45-46)	Age 18-64	1.016, CI: 0.971 - 1.064	1.022, CI: 0.974 - 1.072	1.019, CI: 0.964 - 1.076
		Age 65-74	0.763, CI: 0.629 - 0.926	0.748, CI: 0.622 - 0.9	0.755, CI: 0.598 - 0.953
		Age 75-84	0.985, CI: 0.806 - 1.205	0.977, CI: 0.8 - 1.192	0.941, CI: 0.748 - 1.184
		Age 85+	0.92, CI: 0.608 - 1.393	0.905, CI: 0.613 - 1.335	0.681, CI: 0.403 - 1.153
		All adults	1.013, CI: 0.973 - 1.055	1.018, CI: 0.975 - 1.062	1.022, CI: 0.974 - 1.071
	COPD (J40-44)	Age 18-64	1.056, CI: 1.005 - 1.11	1.069, CI: 1.016 - 1.124	1.07, CI: 1.009 - 1.134
		Age 65-74	1.028, CI: 0.985 - 1.074	1.042, CI: 0.997 - 1.09	1.046, CI: 0.994 - 1.101
		Age 75-84	1.063, CI: 1.018 - 1.111	1.086, CI: 1.038 - 1.135	1.066, CI: 1.01 - 1.125
		Age 85+	1.024, CI: 0.95 - 1.103	1.062, CI: 0.987 - 1.143	1.016, CI: 0.929 - 1.112
		All adults	1.075, CI: 1.049 - 1.102	1.083, CI: 1.056 - 1.111	1.096, CI: 1.065 - 1.128
	Pneumonia (J12-18)	Age 18-64	1.026, CI: 0.988 - 1.066	1.037, CI: 0.997 - 1.078	1.027, CI: 0.983 - 1.073
		Age 65-74	1.039, CI: 0.997 - 1.083	1.049, CI: 1.005 - 1.095	1.025, CI: 0.976 - 1.077
		Age 75-84	1.062, CI: 1.026 - 1.099	1.072, CI: 1.035 - 1.111	1.044, CI: 1 - 1.09
		Age 85+	1.077, CI: 1.04 - 1.116	1.092, CI: 1.052 - 1.133	1.036, CI: 0.993 - 1.081
		All adults	1.07, CI: 1.05 - 1.089	1.076, CI: 1.055 - 1.097	1.057, CI: 1.033 - 1.081
	Respiratory (J00-99)	Age 18-64	1.027, CI: 1.008 - 1.046	1.027, CI: 1.007 - 1.048	1.032, CI: 1.009 - 1.055
		Age 65-74	1.042, CI: 1.017 - 1.068	1.048, CI: 1.022 - 1.075	1.056, CI: 1.026 - 1.088
		Age 75-84	1.071, CI: 1.047 - 1.095	1.08, CI: 1.054 - 1.106	1.065, CI: 1.035 - 1.096
		Age 85+	1.08, CI: 1.054 - 1.107	1.089, CI: 1.062 - 1.118	1.056, CI: 1.023 - 1.089
		All adults	1.059, CI: 1.047 - 1.071	1.06, CI: 1.048 - 1.073	1.06, CI: 1.046 - 1.075
