# Supplementary material for: Extreme heat and cause-specific risk of hospital admission in the adult population in England: a case time series analysis
Source: BMJ Open. 2026 Jun 23;16(6):e105321. doi: 10.1136/bmjopen-2025-105321 (PMC13295835; doi:10.1136/bmjopen-2025-105321)
Supplement: Supplementary file 2 [file bmjopen-2025-105321.reviewer_comments.pdf]

PEER REVIEW HISTORY

BMJ Open publishes all reviews undertaken for accepted manuscripts. Reviewers are asked to complete a checklist review form and are provided with free text boxes to elaborate on their assessment. These free text comments are reproduced below.

ARTICLE DETAILS

Title (Provisional)

Extreme heat and cause-specific risk of hospital admission in the adult population in England: a case time series analysis

Authors

Flower, Gillian; Cole, Rebecca; Mieiro, Luis; Quint, Jennifer K; Gasparrini, Antonio; Masselot, Pierre

---

VERSION 1 - REVIEW

**Reviewer**	**1**
**Name**	**Clougherty , Jane E**
**Affiliation**	**Drexel University**
**Date**	**10-Jul-2025**
**COI**	**None**

Thank you for the opportunity to review this paper. The paper is well done, but have some suggestions to help improve interpretability.

Major comments:

- I find the age groupings very strange. Why the group 0-64? There is such a world of difference between toddlers and near-retirees that I find this group's results uninterpretable (even if the dose-response function is surprisingly similar across smaller age groups therein.) I suggest restricting the paper to adults aged 18+.

- I would like to see more justification of choices made in the statistical models, and some sensitivity-testing. For example, the natural spline includes only one interior knot – how was this choice made? Would any of the curves look meaningfully different allowing more knots? It's not clear to me that what extent the curves reported are affected by this decision, or how this approach may affect the fit for outliers (ie, 99th %ile), which are key to the main comparison.

- Separately, I'd like to see further sensitivity-testing of the DLNM. How would results here compare to, say, using a case-crossover with adjustment for multiple lag days' exposure?

- Other researchers have reported significant effects of heat on injury outcomes and mental health. I know the latter isn't well represented in this dataset, but could injury be included?

Minor comments:

- England is not a particularly hot setting, relatively speaking. Can the authors comment on the relevance of these results to other regions, what we may understand about acclimation (or lack thereof) to heat in England, or to what extent summer temperatures are increasing in England, in relation to other regions?

- Is adjustment for ozone possible? This shouldn't meaningfully change results, as NO2 (which is inversely associated with ozone) is adjusted for, but would be good to confirm.

**Reviewer**	**2**
**Name**	**Hao, Yanbin**
**Affiliation**	**Gannan Medical University**
**Date**	**31-Jul-2025**
**COI**	**None**

This manuscript is well-written and flows smoothly. It clearly describes the purpose and results of the research. I suggest making improvements in the following areas:

The specific analysis model and process description are not particularly clear. There are two stages. Have we only seen the result of the second stage so far? What about the results of the first stage, that is, the results of the regression model in the different LADs?

Have you carried out the meta-combination? The conditions and results of this part of the meta-analysis have not been seen either. What about the heterogeneity? The geographical location maps of different LADs were not seen either.

It would be better to add an equation to describe the analytical model.

What is the lag period for the analysis? How was this chosen （lag0-1）? Are there any sensitivity analysis results available?

Have any of the discovered high or low temperatures shown any protective effect against certain diseases? And some of the exposure response curves show that the risk increases as the temperature rises, and then the highest temperature while shows a protective effect, how can this be explained?

**Reviewer**	**3**
**Name**	**Varghese, Blesson**
**Affiliation**	**The University of Adelaide Faculty of Health and Medical Sciences**

Date 07-Aug-2025

COI None

This manuscript addresses an important and timely topic: the impact of extreme heat on cause-specific hospital admissions in England. The study benefits from a large national dataset, stratified by age, and applies an advanced two-stage modelling approach. However, there are several aspects of the methodology, reporting, and interpretation that would benefit from clarification or expansion. I outline below a series of questions and comments intended to strengthen the manuscript and improve its clarity, robustness, and policy relevance.

• The authors have disaggregated older adults (65+ years) into finer bands (65–74, 75–84, and 85+), which is commendable. However, the age group "0–64" is quite broad, could the authors consider further disaggregating this into finer bands such as 0–14, 15–44, and 45–64 to better capture heterogeneity in heat vulnerability?

• Was sex information available in the dataset? If so, could sex-stratified results be provided?

• Could the authors list the specific conditions examined in the subgroup analyses in the Methods section and provide the corresponding ICD-10 codes used to define them? Why did the authors restrict their cause-specific analysis to the ones they examined and omitted others which have been found to be associated (e.g injuries, maternal and perinatal, mental health etc). If data is available for other causes, suggest that the authors examine other causes as well, please refer to this study: https://pmc.ncbi.nlm.nih.gov/articles/PMC11110655/.

• Could the authors provide the equation or a schematic of the first-stage model to clarify how the regression was specified?

• Why were PM2.5 and NO_2 included only linearly over lag 0–1? Was any sensitivity analysis conducted to test alternative lag structures or non-linear terms?

• How did the authors decide on placing spline knots at the 50th and 90th percentiles in the exposure–response function? Were other knot placements tested?

• Why was a lag period of only 3 days used, and were longer lags (e.g., 5–7 days) tested, given that heat effects may persist for several days for some health outcomes?

• Why was the 99th percentile compared to the 50th percentile for estimating relative risks? Does the 50th percentile represent the minimum morbidity temperature (MMT), or was it selected as a reference for another reason?

• The methods indicate that LSOA-specific time series were modelled within LAD-specific quasi-Poisson regression models. Could the authors clarify whether this structure allows for estimation of LAD-specific or LSOA-level relative risks? If so, were spatial patterns of heat-related risk explored or visualised? Presenting LAD- or LSOA-level estimates (e.g., via

choropleth maps) could highlight geographic variability in vulnerability and improve the policy relevance of the findings.

• Can the authors detail whether any sensitivity analyses were conducted to confirm the robustness of their model specifications (e.g., lag structure, spline placement, covariates)? If so, please provide such results in the appendix.

• The manuscript states that seasonality was adjusted using a natural spline with 4 degrees of freedom. Could the authors clarify whether this spline was applied to day of year or calendar time, and how the degrees of freedom were selected? Given the 12-year study period, did the authors consider allowing for year-specific seasonal effects (e.g., interaction with year indicators or stratified seasonal terms)?

• Given the use of LSOA-level data, did the authors consider including any area-level indicators of vulnerability, such as the Index of Multiple Deprivation (IMD), urban–rural classification, vegetation, land surface temperature, climate zone, or population density? Incorporating such factors would allow exploration of differential vulnerability to heat across socioeconomic or spatial lines, which is highly relevant to public health targeting and climate adaptation. Even simple stratification or interaction analysis with IMD quintiles and other variables (e.g., high vs. low levels) and presenting them in a forest plot could offer valuable insight.

• The authors acknowledge that they report country-wide estimates and did not assess local differences in risk, but given the availability of LSOA-level data and LAD-specific models, this omission is surprising. This should be acknowledged more explicitly as a limitation. If spatial analyses are not feasible, please explain why.

• There is no mention of exposure misclassification, which is relevant in time-series studies using area-level temperatures. The authors should briefly acknowledge that not all residents within a LAD experience the same exposure (e.g., due to urban heat islands or proximity to green space or coastlines).

• The terms “hospital admissions” and “hospital stays” are used interchangeably. Unless length of stay is specifically analyzed, “hospital admissions” should be used consistently throughout to avoid confusion.

• While some effect estimates (RRs with 95% CIs) are reported, others are described qualitatively (e.g., “no clear evidence”). Could the authors clarify what is meant by "no clear evidence"? .

• The results refer to the RR for the 99th percentile versus the 50th percentile, but it’s not clearly stated whether the 50th percentile represents the minimum morbidity temperature (MMT) or simply the median. This should be clarified in both the Methods and Results sections to aid interpretation.

• While relative risks are useful for quantifying associations, the public health relevance of the findings would be significantly strengthened by estimating the attributable fraction (AF)

and attributable number (AN) of hospital admissions due to extreme heat. These measures provide a clearer sense of burden and are more directly interpretable for health service planning and policy-making. I recommend including these in the revised version.

• In addition to estimating attributable admissions, would the authors consider providing a basic estimate of the healthcare cost burden associated with heat-related hospitalisations? Using publicly available NHS reference costs per admission and multiplying by the attributable number of cases by cause would provide a national-level approximation of economic impact which would enhance the relevance of the study for public health and economic planning. I refer the authors to this study for a comparable approach: https://www.sciencedirect.com/science/article/pii/S0048969724081798#s0010

• Given the observed associations and the availability of national-level data, would the authors consider providing projections of future heat-related hospitalisation burden under plausible climate change scenarios? Incorporating projected temperature increases and demographic changes (e.g., population ageing) could enhance the policy relevance of the study and provide valuable insight for long-term healthcare and public health planning.

Lastly, based on the changes made to the main part of the manuscript in line with above suggestions, please review and revise the abstract accordingly.

VERSION 1 - AUTHOR RESPONSE

Reviewer 1

Thank you for the opportunity to review this paper. The paper is well done, but I have some suggestions to help improve interpretability.

Major comments:

1.1) *I find the age groupings very strange. Why the group 0-64? There is such a world of difference between toddlers and near-retirees that I find this group's results uninterpretable (even if the dose-response function is surprisingly similar across smaller age groups therein.) I suggest restricting the paper to adults aged 18+.*

The authors acknowledge that this is a valid concern. The original age groups were chosen to provide adequate discrimination among older adults, who form the majority of hospital admissions for most diagnoses, whilst ensuring sufficient statistical power. However, we do agree with the restriction to adults for better interpretability. A revised age grouping of ages 18-64, 65-74, 75-84 and 85+ has now been used. Unfortunately, additional segmentation of the youngest age group (ages 18-64) reduces the number of cases too much and therefore does not allow for sufficient statistical power

1.2) *I would like to see more justification of choices made in the statistical models, and some sensitivity-testing. For example, the natural spline includes only one interior knot – how was this choice made? Would any of the curves look meaningfully different allowing more knots? It's not clear to me that what extent the curves*

reported are affected by this decision, or how this approach may affect the fit for outliers (ie, 99th %ile), which are key to the main comparison.

Thank you for your comment. The knots used are typical of the heat-related health extensive literature, capturing accelerating risk (knot at 90th percentile) and almost immediate effect (knot at lag 1) at high percentiles of temperature. We have clarified this aspect in paragraphs one and two of the Statistical Methods section on page 5 of the manuscript. However, we do agree that most of this literature refers to mortality rather than hospital admissions, therefore, sensitivity analyses of alternative parameterisations are now included as Supplementary Material, where it can be seen that results closely resemble those of the main analysis. We have added sentences about these sensitivity analyses under 'Temperature-related risk of admission' on page 7 in the Results section, at the end of the Methods section on page 6.

1.3) *Separately, I'd like to see further sensitivity-testing of the DLNM. How would results here compare to, say, using a case-crossover with adjustment for multiple lag days' exposure?*

Our analysis uses a case time series design which generalises the case-crossover design by allowing monthly stratification via conditional Poisson models. The DLNM term is included in this design and refers to the functional assumptions of the risk, specifically, it accounts for non-linear and delayed effects, which have been shown to be less prone to bias compared to lag adjustment via stratification or moving averages. (Please see the following paper for additional information: https://doi.org/10.1097/EDE.0000000000000533). We have included a sensitivity analysis that extended the lag period to five days (please also refer to Comment 1.2 above).

1.4) *Other researchers have reported significant effects of heat on injury outcomes and mental health. I know the latter isn't well represented in this dataset, but could injury be included?*

The dataset we used for our analysis, the Admitted Patient Care (APC) tables, does not capture all types of hospital attendance. Attendances due to injury are primarily recorded via the Emergency Care Dataset (ECDS) while psychiatric ward admissions are primarily captured in the Mental Health Dataset (MHDS). For this reason, inclusion of these conditions and presentation of results alongside the other health conditions, would result in an underpowered study as well as potential selection biases. The scope of the APC tables is a limitation we acknowledge in the Discussion and Methods sections of the manuscript (please refer to paragraph 2 of the Methods section on page 4, and paragraph 4 of the Discussion section on page 10 of the manuscript).

Minor comments:

1.5) *England is not a particularly hot setting, relatively speaking. Can the authors comment on the relevance of these results to other regions, what we may understand*

about acclimation (or lack thereof) to heat in England, or to what extent summer temperatures are increasing in England, in relation to other regions?

This is an interesting question. We did not attempt to measure the overall trend in summer temperatures, and the extent to which temperatures are increasing in England as part of this research. There are, however, very relevant papers cited in the Introduction section of our manuscript, particularly the 2023 State of the UK Climate report, that mentions a trebling of very hot days since 1961 to 1990. Due to the varying nature of healthcare systems in different settings, each of which will have their own data collection mechanisms and thresholds for admission for different health conditions, findings from this research are not considered generalisable to alternative settings. This is a limitation we acknowledge in paragraph 4 of the Discussion section of the manuscript (quoted here for reference "*Due to the complex nature of healthcare systems both in the UK and globally, findings from this study are not directly comparable to populations in other countries, which may present with very different exposure patterns, socio-demographic characteristics, and thresholds for hospital admission.")*.

1.6) Is adjustment for ozone possible? This shouldn't meaningfully change results, as NO2 (which is inversely associated with ozone) is adjusted for, but would be good to confirm.

We agree that it would be relevant. Unfortunately, UK-wide ozone data of sufficient quality was not available for inclusion in this analysis. We acknowledge this limitation in paragraph 4 of the Discussion.

Reviewer 2

This manuscript is well-written and flows smoothly. It clearly describes the purpose and results of the research. I suggest making improvements in the following areas:

2.1) The specific analysis model and process description are not particularly clear. There are two stages. Have we only seen the result of the second stage so far? What about the results of the first stage, that is, the results of the regression model in the different LADs?

Thank you for your comments. We acknowledge that the description of the statistical methods used in the analysis could be clearer. We have amended the Methods section of the manuscript to provide clarification, including the addition of a formula to represent the first stage of the analysis.
The focus of our research was to provide England-level effect estimates for each cause of admission, by age, whilst adjusting for local variation in exposure patterns. Given the relatively low numbers in some LADs and for some diagnoses, LAD-level results are highly uncertain to be reported as such, which is why we decided to focus on the (much more stable) overall effect. Also note that the paper covers many different diagnoses and additional spatial disaggregation of these results would have created a very large amount of material to include in this paper. We chose to

maintain a more concise focus for this research, and report only the final England-level results by age and diagnosis.

2.2) *Have you carried out the meta-combination? The conditions and results of this part of the meta-analysis have not been seen either. What about the heterogeneity? The geographical location maps of different LADs were not seen either. It would be better to add an equation to describe the analytical model.*

Thank you for your comments. We acknowledge that the Methods section required clarification. We have amended this according, including the addition of a formula to illustrate the first stage model. Please see our response to item 2.1 for additional information relating to the reporting of results. Given the high number of different hospitalisation causes assessed in our analysis, we chose to focus the results on the pooled effects across England. The full mapping and analysis of heterogeneity for all the causes would be difficult to report in a single article, and will hopefully be carried out for each specific cause in future research. We acknowledge this limitation paragraph 4 of the Discussion (page 10).

2.3) *What is the lag period for the analysis? How was this chosen (lag0-1)?*

Our research focused on the relatively short-term effects of heat in the summer months. We included delayed effects of temperature up to 3 days (lag 0-3). We included adjustment for air pollution ($PM_{2.5}$ and NO_2) at the time of admission (lag 0-1). This is a standard specification that usually captures well the short-term vulnerability to $PM_{2.5}$.

2.4) *Are there any sensitivity analysis results available?*
Results of our sensitivity analyses have now been included as Supplementary Material (B). This includes two alternative parameterisations:

I. An adjustment to the temperature-response function to use 1 knot placed at the 75th percentile (our main analysis uses 2 knots placed at the 50th and 90th temperature percentile), and
II. An extension of the lag-period to 5 days with 1 internal knot (our main analysis uses a lag-period of 3 days, with 1 internal knot)

These results are shown alongside estimates from the main analysis and show that the conclusions still hold under these different specifications.

2.5) *Have any of the discovered high or low temperatures shown any protective effect against certain diseases? And some of the exposure response curves show that the risk increases as the temperature rises, and then the highest temperature while shows a protective effect, how can this be explained?*

Thank you for your comments. We did find results suggesting a lower or negative relative risk of admission at the 99th temperature percentile (relative to the 50th), for some cardiovascular health conditions. In these instances, we are cautious of interpreting these results as evidence of a 'protective' effect of extreme heat. This was because it is plausible that lower estimates are caused by mortality prior to

hospitalisation, rather than a true beneficial effect of heat. Please refer to paragraph 4 of the Discussion section on page 10 of the manuscript for additional commentary relating to the cardiovascular results (extracts quoted below for reference).

"A possible explanation is pre-hospitalisation mortality, either in the community or during emergency department attendance. We would expect pre-admission mortality to decrease the perceived risk of hospitalisation, particularly for acute events such as MI or stroke, and among older patients where mortality risk is greater, both of which were observed in this study."

"It is worth noting that patients experiencing suspected MI or stroke are prioritised for ambulance response, so it is plausible that a higher proportion of these patients are managed via emergency department care."

We believe this area warrants further research.

Reviewer 3

This manuscript addresses an important and timely topic: the impact of extreme heat on cause-specific hospital admissions in England. The study benefits from a large national dataset, stratified by age, and applies an advanced two-stage modelling approach. However, there are several aspects of the methodology, reporting, and interpretation that would benefit from clarification or expansion. I outline below a series of questions and comments intended to strengthen the manuscript and improve its clarity, robustness, and policy relevance.

3.1) The authors have disaggregated older adults (65+ years) into finer bands (65–74, 75–84, and 85+), which is commendable. However, the age group "0–64" is quite broad, could the authors consider further disaggregating this into finer bands such as 0–14, 15–44, and 45–64 to better capture heterogeneity in heat vulnerability?

The authors acknowledge that this is a valid concern. The original age groups were chosen to provide adequate discrimination among older adults, who form the majority of hospital admissions for most diagnoses, whilst ensuring sufficient statistical power. Therefore, it is difficult to further stratify the 0-64 age group due to potentially very unstable estimates. However, we do agree that it is relevant to separate children from adults and have decided to apply a revised age grouping excluding admissions relating to patients under 18 years of age.

3.2) Was sex information available in the dataset? If so, could sex-stratified results be provided?

Our research focused primarily on understanding age-related variation in relative risk of hospitalization, across a range of health conditions. Unfortunately, further disaggregation of each health condition by age and sex would substantially reduce the number of admissions in each sub-group. The inclusion of sex would therefore necessitate the aggregation or exclusion of some health conditions and/or age

groups. While this is a valid research aim, this was not the intended focus of this work. We chose to focus on age across a broad range of health conditions.

3.3) *Could the authors list the specific conditions examined in the subgroup analyses in the Methods section and provide the corresponding ICD-10 codes used to define them? Why did the authors restrict their cause-specific analysis to the ones they examined and omitted others which have been found to be associated (e.g injuries, maternal and perinatal, mental health etc). If data is available for other causes, suggest that the authors examine other causes as well, please refer to this study: https://pmc.ncbi.nlm.nih.gov/articles/PMC11110655/.*

Thank you for your comments. Please note we have included ICD codes for each grouping in Table 1 of the manuscript. We created our dataset using the Admitted Patient Care tables from NHS Digital. A limitation of this dataset is that it does not include records for all types of NHS attendance. Therefore, we decided to limit the analysis to the diagnoses that are well represented in the available dataset. In particular, these tables do not include all information relating to Emergency Department visits, admissions to psychiatric wards, maternity service usage or inpatient stays during pregnancy. Information relating to attendances for these conditions is primarily recorded in alternative NHS databases, specifically the Emergency Care Dataset (ECDS), Mental Health Services Dataset (MHSDS) and the Maternity Services Dataset (MSDS). For this reason, inclusion of effect estimates for these conditions, derived solely from the APC tables available to us, was considered misleading and would likely underestimate the true effect of heat on these conditions. This is a limitation we acknowledge in the Methods and Discussion sections of the manuscript.

3.4) *Could the authors provide the equation or a schematic of the first-stage model to clarify how the regression was specified?*

Yes, we acknowledge that the Methods section of the manuscript required further clarification. We have now amended accordingly, including the addition of a formula to illustrate the first stage model used in the analysis.

3.5) *Why were PM2.5 and NO_2 included only linearly over lag 0–1? Was any sensitivity analysis conducted to test alternative lag structures or non-linear terms?*

The choice of lag structure for $PM_{2.5}$ and NO_2 was based on consistency with existing literature on short-term effects. Considering nonlinear terms for confounders would result in unwarranted additional model complexity with little benefit for control as air pollutants are almost always modelled linearly in time series analyses. Please see examples provided below: https://doi.org/10.1186/1476-069X-13-38, https://doi.org/10.1289/ehp.1206151, https://doi.org/10.1136/bmj-2023-076322.

3.6) *How did the authors decide on placing spline knots at the 50th and 90th percentiles in the exposure–response function? Were other knot placements tested?*

The knots used are typical of the heat-related health extensive literature, capturing accelerating risk (knot at 90th percentile) and almost immediate effect (knot at lag 1) at high percentiles of temperature. We have amended wording in the manuscript to clarify this aspect (in the first paragraph of the Statistical Methods section on page 6). However, we do agree that most of this literature refers to mortality rather than hospital admissions, therefore, sensitivity analyses of alternative parameterisations are now included as Supplementary Material, showing little differences with the main results.

3.7) *Why was a lag period of only 3 days used, and were longer lags (e.g., 5–7 days) tested, given that heat effects may persist for several days for some health outcomes?*

We used a lag period of 0-3 days mainly for two reasons: i) the effect of heat is often immediate and previous studies have shown that most of the impact is concentrated over two days, and ii) longer lags imply more complex models which add to the power concerns we have expressed in previous replies. We did however check the sensitivity of the analysis to alternative lag structures, including a 0-5 day lag. Further details of this sensitivity analysis can now be found in our Supplementary Material.

3.8) *Why was the 99th percentile compared to the 50th percentile for estimating relative risks? Does the 50th percentile represent the minimum morbidity temperature (MMT), or was it selected as a reference for another reason?*

The 50th temperature percentile was chosen as a standardised interpretation across conditions given the wide heterogeneity in MMT between conditions. This was used to aid comparability instead of reporting multiple MMTs for each age group and condition. It is also worth noting that in some instances, there wasn't a clear single temperature, but rather a range of temperatures representing low morbidity risk. We have amended wording in the Methods section of the manuscript to clarify this point (please refer to the last paragraph on page 6 of the manuscript).

3.9) *The methods indicate that LSOA-specific time series were modelled within LAD-specific quasi-Poisson regression models. Could the authors clarify whether this structure allows for estimation of LAD-specific or LSOA-level relative risks? If so, were spatial patterns of heat-related risk explored or visualised? Presenting LAD- or LSOA-level estimates (e.g., via choropleth maps) could highlight geographic variability in vulnerability and improve the policy relevance of the findings.*

The focus of our research was to provide England-level effect estimates by age, whilst adjusting for local variation in exposure patterns. While the modelling framework does allow quantification of LAD-level relative risks and spatial exploration of attributable hospital admissions the low number of cases in some LADs for some diagnoses resulted in uncertain LAD level estimates which would not provide informative spatial patterns hence our decision to focus on the pooled effect. This would additionally have created a large volume of results for inclusion in one paper;

we instead chose to keep a narrower focus on England-level results by age and diagnosis.

3.10) *Can the authors detail whether any sensitivity analyses were conducted to confirm the robustness of their model specifications (e.g., lag structure, spline placement, covariates)? If so, please provide such results in the appendix.*

Results of our sensitivity analyses have now been included as Supplementary Material. This includes two alternative parameterisations:

I. An adjustment to the temperature-response function to use 1 knot placed at the 75th percentile (our main analysis uses 2 knots placed at the 50th and 90th temperature percentile), and

II. An extension of the lag-period to 5 days with 1 internal knot (our main analysis uses a lag-period of 3 days, with 1 internal knot)

These results are shown alongside estimates from the main analysis and show that our conclusion remain unchanged with alternative specifications.

3.11) *The manuscript states that seasonality was adjusted using a natural spline with 4 degrees of freedom. Could the authors clarify whether this spline was applied to day of year or calendar time, and how the degrees of freedom were selected? Given the 12-year study period, did the authors consider allowing for year-specific seasonal effects (e.g., interaction with year indicators or stratified seasonal terms)?*

Thank you for highlighting this. We have amended the Methods section of the manuscript to provide additional clarification regarding adjustment for temporal trends.

We applied two levels of temporal control. (1) The first as a LAD-level adjustment for seasonality for each health condition, using a natural spline with 4 degrees of freedom per year, applied to day of the year. (2) In addition to this LAD-level adjustment, we applied a strict control for seasonality and long-term trends at LSOA level, through a stratification in the conditional Poisson model by month/year.

Collectively, these adjustments were considered to provide extensive control for temporal trends, whilst allowing within-year/month and within-LSOA variability for each condition. Weekday and public holiday effects were also controlled for using indicator variables.

3.12) *Given the use of LSOA-level data, did the authors consider including any area-level indicators of vulnerability, such as the Index of Multiple Deprivation (IMD), urban–rural classification, vegetation, land surface temperature, climate zone, or population density? Incorporating such factors would allow exploration of differential vulnerability to heat across socioeconomic or spatial lines, which is highly relevant to public health targeting and climate adaptation. Even simple stratification or interaction analysis with IMD quintiles and other variables (e.g., high vs. low levels) and presenting them in a forest plot could offer valuable insight.*

The primary aim of our research was to facilitate reporting of England-level relative risks, whilst adjusting for location variation in exposure. While we acknowledge that this is a valid area for future research, we did not attempt to quantify additional factors influencing vulnerability beyond age, as part of this study. We also noted the additional complexity this would introduce when comparing results across multiple health conditions as well as uncertainty of LAD-level results.

3.13) *The authors acknowledge that they report country-wide estimates and did not assess local differences in risk, but given the availability of LSOA-level data and LAD-specific models, this omission is surprising. This should be acknowledged more explicitly as a limitation. If spatial analyses are not feasible, please explain why.*

The focus of our research was to provide England-level effect estimates by age, whilst adjusting for local variation in exposure patterns. This was our reason for reporting only the overall England-level results. While the modelling framework does allow quantification of LAD-level relative risks and spatial exploration of attributable hospital admissions such results would be too unstable for several diagnostics to provide informative results.

3.14) *There is no mention of exposure misclassification, which is relevant in time-series studies using area-level temperatures. The authors should briefly acknowledge that not all residents within a LAD experience the same exposure (e.g., due to urban heat islands or proximity to green space or coastlines).*

Please note that the case time series study design assigned exposures at the LSOA level, rather than at LAD-level. LSOAs being generally small, this reduces the risk of exposure misclassification. Note that the main risk of exposure misclassification in these settings is of increased variability of estimates rather than bias, which will be limited here given the small size of areas. For additional information, please refer to the following article: https://doi.org/10.1093/oxfordjournals.aje.a115761.

3.15) *The terms "hospital admissions" and "hospital stays" are used interchangeably. Unless length of stay is specifically analyzed, "hospital admissions" should be used consistently throughout to avoid confusion.*

Thank you for highlighting this. We have amended the wording in the manuscript to consistently refer to 'hospital admissions' rather than using the two terms interchangeably.

3.16) *While some effect estimates (RRs with 95% CIs) are reported, others are described qualitatively (e.g., "no clear evidence"). Could the authors clarify what is meant by "no clear evidence"?*
The authors considered instances where the effect estimates and associated confidence intervals excluded the reference point (a relative risk of 1, i.e. the risk associated with the median temperature) as evidence of heat-related risk of hospital admission. Where this was not the case, and relative risks and their confidence intervals included the reference point, this was considered as providing no evidence

of an increased or decreased risk of hospital admission, relative to the median temperature. However, we have now added the corresponding RRs in these cases as well for better consistency with the rest of the reporting (0.97, CI: 0.97-1.00, and 0.98, CI: 0.96 – 1.01 for MI and stroke respectively).

3.17) *The results refer to the RR for the 99th percentile versus the 50th percentile, but it's not clearly stated whether the 50th percentile represents the minimum morbidity temperature (MMT) or simply the median. This should be clarified in both the Methods and Results sections to aid interpretation.*

Thank you for highlighting this. We have amended wording in the Methods section of the manuscript to clarify this point. The 50th temperature percentile (median) was chosen as the reference point for calculating the relative risks in order to allow more interpretable comparison between health conditions. This choice was additionally motivated by the difficulty to define a clear MMT in some instances (see curves in appendix).

3.18) *While relative risks are useful for quantifying associations, the public health relevance of the findings would be significantly strengthened by estimating the attributable fraction (AF) and attributable number (AN) of hospital admissions due to extreme heat. These measures provide a clearer sense of burden and are more directly interpretable for health service planning and policy-making. I recommend including these in the revised version.*

Thank you for your comments. While we agree that the reporting of attributable hospital admissions is a valid and helpful tool to facilitate a greater understanding of the health burden associated with heat, the inclusion in this paper was not considered appropriate. Indeed, we think it would be inaccurate to use an overall exposure-response function to compute these measures, while the uncertainty of LAD level results makes the estimation of the burden more difficult. We believe it would be more appropriate to tackle this part in future research with augmented datasets and improved methodologies.

3.19) *In addition to estimating attributable admissions, would the authors consider providing a basic estimate of the healthcare cost burden associated with heat-related hospitalisations? Using publicly available NHS reference costs per admission and multiplying by the attributable number of cases by cause would provide a national-level approximation of economic impact which would enhance the relevance of the study for public health and economic planning. I refer the authors to this study for a comparable approach:*
https://www.sciencedirect.com/science/article/pii/S0048969724081798#s0010

This is an interesting point. We agree that this would be a valid extension to our research, but given the complex and varied factors influencing cost attributable to individual hospital stays, this was considered beyond the scope of our research and primary aims of understanding hospitalisation risk. This is something we would like to

consider as future research, potentially adjusting for key factors such as repeat attendances, complexity of clinical presentation and length of stay.

3.20) *Given the observed associations and the availability of national-level data, would the authors consider providing projections of future heat-related hospitalisation burden under plausible climate change scenarios? Incorporating projected temperature increases and demographic changes (e.g., population ageing) could enhance the policy relevance of the study and provide valuable insight for long-term healthcare and public health planning.*

We agree that this is an important point. An understanding of hospitalization risk under climate change scenarios is a valid extension to this work, and something we would be interested to explore as an area of further research. Unfortunately, in the same fashion as the economic valuation suggested above, this was considered beyond the scope of this study.

3.21) *Lastly, based on the changes made to the main part of the manuscript in line with above suggestions, please review and revise the abstract accordingly.*

Yes, we have amended the Abstract accordingly.

VERSION 2 - REVIEW

**Reviewer**	**1**
**Name**	**Clougherty , Jane E**
**Affiliation**	**Drexel University**
**Date**	**26-Nov-2025**
**COI**	

Thank you for the opportunity to re-review this manuscript.

The authors have thoughtfully responded to all of my comments. I appreciate that they limited the analysis to adults over age 18, which makes for a more interpretable set of results, and compared effects across adult age groups.

I appreciate the more extensive description of statistical methods, the choice of statistical methods, and sensitivity tests provided. I also appreciate the acknowledgement of some key limitations of the analysis in the Discussion – including some outcomes not well captured in this dataset, inability to adjust for ozone exposures, and the uncertain relevance of results for ‘high’ heat days in England to higher heat exposures or impacts in other parts of the world.

**Reviewer**	**2**
**Name**	**Hao, Yanbin**
**Affiliation**	**Gannan Medical University**
**Date**	**21-Nov-2025**
**COI**	

I think the authors have seriously considered and responded to the reviewers' comments.

**Reviewer**	**3**
**Name**	**Varghese, Blesson**
**Affiliation**	**The University of Adelaide Faculty of Health and Medical Sciences**
**Date**	**24-Nov-2025**
**COI**	

The authors have responded to all the points that I raised earlier. However, I have few more comments for their consideration before recommending this manuscript for publication.

1. Please clarify that the APC dataset excludes A&E attendances that do not lead to admission. Presently it says APC excludes emergency department attendances, but technically A&E attendances are only excluded when they don't result in an admission. Adding this clarification will improve accuracy.

2. Thank you for revising the age groups as per the comment in 3.1. The new breakdown (18–64, 65–74, 75–84, 85+) is more informative than the previous 0–64 / 65+ split. However, since you have access to the full dataset and have already introduced finer age categorisation, it would be important to clarify why ages 0–17 was excluded. Children constitute a meaningful proportion of unplanned admissions, and there is an established evidence base on paediatric heat vulnerability. For example, the recent New Zealand case time-series study analysed 647,000 paediatric admissions (aged under 5 years) at SA2 level (population of 2,000-4,000 residents), despite New Zealand having a much smaller population than the UK. This suggests that a UK-wide analysis should also be feasible. I strongly recommend including the 0–17 group unless there is a strong methodological rationale not to. If inclusion is not feasible, please provide a clear justification and acknowledge this in the limitation section. Please also clarify whether the Figures and tables in the appendix for total or all ages includes everyone or is it those aged 18+. Finally, recommend adjusting the title to reflect the study population (e.g., '…in adults in England') as the current title maybe misleading as it implies population-wide coverage.

3. Thank you for the response to 3.3. However, my original request was for overall sex-stratified estimates (e.g., separate male/female associations), not age × sex × condition stratification. This would not create small-cell issues and would strengthen the analysis. If this is not feasible, please provide a justification specific to simple sex stratification.

4. Thank you for the clarifications in 3.9, 3.12 and 3.13. However, these responses relate to a common issue: the absence of any spatial or vulnerability analysis despite the availability of LSOA-level data and LAD-specific first-stage models. My original comments were not limited to diagnosis-specific mapping. To clarify, the modelling framework you describe—a two-stage design with LAD-specific quasi-Poisson models and a second-stage random-effects multivariate meta-analysis—naturally supports the exploration of spatial variation in heat-related risk. There are at least two feasible and widely used options in the DLNM literature: (i) estimating LAD-level relative risks for a combined heat-sensitive outcome (pooling all included diagnoses), which would substantially increase case counts and improve stability, enabling the production of a single choropleth similar to that used by Gasparrini et al. for mortality; and (ii) incorporating area-level indicators such as IMD quintiles, urban–rural classification, vegetation, land surface temperature, or population density in the second-stage meta-regression. These approaches do not require diagnosis-specific stratification and would provide valuable insight into differential vulnerability, a key issue for public health and climate adaptation. If the authors wish to retain a national-only focus, I recommend explicitly acknowledging the omission of any spatial or vulnerability analysis as a limitation and briefly justifying why these feasible extensions were not undertaken, given the modelling framework and data available.

5. Appreciate the clarification in 3.14. Using LSOA-level temperature does indeed reduce spatial exposure misclassification compared with LAD-level assignment. However, some degree of exposure misclassification is unavoidable in ecological time-series studies, because individual-level exposure cannot be captured (e.g., differences in time spent indoors/outdoors, commuting patterns, building characteristics, and microclimatic variation within LSOAs). Even if such misclassification primarily increases variance rather than bias, it would still be appropriate to briefly acknowledge this as a limitation in the manuscript, as is standard in environmental epidemiology.

6. Thank you for the response in 3.18. However, estimating the attributable fraction (AF) and attributable number (AN) at the national level is standard in multi-location DLNM studies. The England-wide pooled exposure–response function you already compute is sufficient to derive a national AF/AN, and I strongly suggest doing so would substantially strengthen the policy relevance of the paper. Policymakers generally rely on AF/AN to quantify burden, as these measures translate relative risks into an interpretable number of additional hospital admissions attributable to heat. If the authors strongly prefer not to include these estimates, I recommend acknowledging this as a limitation and providing a clearer justification, given their central importance for public health planning.

7. Thank you for the response in 3.19 and 3.20. I accept that cost estimation and climate-change projections are beyond the current scope and appreciate the authors identifying these as valuable areas for future work. I suggest briefly noting these potential extensions in the discussion.

8. The manuscript states that statistical code is available at the referenced GitHub repository (github.com/gmflower/UK-TRH), but the repository currently contains no code/materials. Please correct the link and/or upload the full analysis code. If the repository is private or not yet public, please make it publicly accessible prior to publication.

VERSION 2 - AUTHOR RESPONSE

Reviewer 3

The authors have responded to all the points that I raised earlier. However, I have few more comments for their consideration before recommending this manuscript for publication.

1) *Please clarify that the APC dataset excludes A&E attendances that do not lead to admission. Presently it says APC excludes emergency department attendances, but technically A&E attendances are only excluded when they don't result in an admission. Adding this clarification will improve accuracy.*

   Thank you for highlighting this. Your interpretation is correct; emergency department attendances are not included in our dataset, but patients visiting an emergency department who are subsequently admitted to a ward are included in our analysis. We use the term 'attendances' to make this distinction. We have now acknowledged this more explicitly in paragraph 2 of the Methods section in the revised manuscript.

2) *Thank you for revising the age groups as per the comment in 3.1. The new breakdown (18–64, 65–74, 75–84, 85+) is more informative than the previous 0–64 / 65+ split. However, since you have access to the full dataset and have already introduced finer age categorisation, it would be important to clarify why ages 0–17 was excluded. Children constitute a meaningful proportion of unplanned admissions, and there is an established evidence base on paediatric heat vulnerability. For example, the recent New Zealand case time-series study analysed 647,000 paediatric admissions (aged under 5 years) at SA2 level (population of 2,000-4,000 residents), despite New Zealand having a much smaller population than the UK. This suggests that a UK-wide analysis should also be feasible. I strongly recommend including the 0–17 group unless there is a strong methodological rationale not to. If inclusion is not feasible, please provide a clear justification and acknowledge this in the limitation section. Please also clarify whether the Figures and tables in the appendix for total or all ages includes everyone or is it those aged 18+. Finally, recommend adjusting the title to reflect the study population (e.g., '…in adults in England') as the current title maybe misleading as it implies population-wide coverage.*

Thank you for your comments. We recognise the importance of distinguishing ages 0-17 from the adult population. However, as Reviewer 1 (see above) acknowledged, there are important distinctions between adult and paediatric interactions with the health service, as well as very different profiles of vulnerability. It is for these reasons that the authors believe an appropriate approach would be to address these issues via a future distinct analysis focused primarily on ages 0-17 years.
Please note that the scope of the analysis, as now restricted to ages 18 and older, is acknowledged in the Abstract. We have also now amended the study title to clarify this issue.

3) *Thank you for the response to 3.3. However, my original request was for overall sex-stratified estimates (e.g., separate male/female associations), not age × sex × condition stratification. This would not create small-cell issues and would strengthen the analysis. If this is not feasible, please provide a justification specific to simple sex stratification.*

   We acknowledge that this would be a valid area of research to consider sex-related differences in risk. However, sex-specific analyses were not part of the original analysis plan, and the assessment of differences in risks of heat-related hospital admissions between sexes deserves more space and elaboration than it is possible to allocate in this manuscript, which explicitly focuses on comparison between admission causes.

4) *Thank you for the clarifications in 3.9, 3.12 and 3.13. However, these responses relate to a common issue: the absence of any spatial or vulnerability analysis despite the availability of LSOA-level data and LAD-specific first-stage models. My original comments were not limited to diagnosis-specific mapping. To clarify, the modelling framework you describe—a two-stage design with LAD-specific quasi-Poisson models and a second-stage random-effects multivariate meta-analysis—naturally supports the exploration of spatial variation in heat-related risk. There are at least two feasible and widely used options in the DLNM literature: (i) estimating LAD-level relative risks for a combined heat-sensitive outcome (pooling all included diagnoses), which would substantially increase case counts and improve stability, enabling the production of a single choropleth similar to that used by Gasparrini et al. for mortality; and (ii) incorporating area-level indicators such as IMD quintiles, urban–rural classification, vegetation, land surface temperature, or population density in the second-stage meta-regression. These approaches do not require diagnosis-specific stratification and would provide valuable insight into differential vulnerability, a key issue for public health and climate adaptation. If the authors wish to retain a national-only focus, I recommend explicitly acknowledging the omission of any spatial or vulnerability analysis as a limitation and briefly justifying why these feasible extensions were not undertaken, given the modelling framework and data available.*

   Thank you for your comments. The authors agree that the statistical framework they developed and applied in a previous analysis on temperature-related risks for all-cause mortality does facilitate both (a) spatial exploration of differences in risk, and (b) incorporation of indicators of vulnerability, such as deprivation. However, there are

several reasons why replicating the same analysis using this framework was not appropriate in this situation:

- As stated above, the focus of this analysis is on the comparison of risks of hospital admission for different causes. This requires the definition of summary estimates that can be easily interpreted and compared across causes. The analysis of geographical patterns of risks and the assessment of vulnerability factors are beyond the scope of the original analysis plan and would divert attention from the main focus of the manuscript. The proposed approach would also rely on an implicit assumption that factors influencing vulnerability are consistent across diagnoses, that is not necessarily appropriate.
- In addition, the type of analysis presented by the authors in their previous article (Gasparrini et al, Lancet Planetary Health 2022) presented a wide range of summary outputs, including detailed risk maps and stratification by age, urban vs rural areas, and deprivation classes. This is appropriate for an analysis focused on a single health outcome, namely all-cause mortality, but it is not feasible when presenting results for a long list of different outcomes, as in this analysis.
- More importantly, the assessment of cause-specific hospital admissions presents additional complexities compared to all-cause mortality, given the more nuanced definition of the health outcomes. One noteworthy example is the set of results presented here for cardiovascular admissions, which show unexpected results, with negative risks associated with heat. Pooling across diagnosis specific estimates to obtain overall heat related relative risks of hospital admission overlooks some of these important nuances. These results deserve more elaboration and discussion than the more straightforward estimates obtained for all-cause mortality, and therefore necessitate the definition of simpler effect summaries and a more focused analysis.

We believe therefore that the type of analysis we produced for all-cause mortality can be repeated in future work focused on specific admission causes, while this contribution should focus on a direct and simpler comparison between multiple causes of hospital admissions.

5) *Appreciate the clarification in 3.14. Using LSOA-level temperature does indeed reduce spatial exposure misclassification compared with LAD-level assignment. However, some degree of exposure misclassification is unavoidable in ecological time-series studies, because individual-level exposure cannot be captured (e.g., differences in time spent indoors/outdoors, commuting patterns, building characteristics, and microclimatic variation within LSOAs). Even if such misclassification primarily increases variance rather than bias, it would still be appropriate to briefly acknowledge this as a limitation in the manuscript, as is standard in environmental epidemiology.*

Thank you for your comments. Whilst we agree that some degree of misclassification is almost inevitable in environmental studies using residence as a method of

exposure assignment, we believe that the use of LSOA-level assignment is in fact an important strength of this study, compared to most of the existing literature that often relies on a more aggregated geographical level of exposure assignment.

6) *Thank you for the response in 3.18. However, estimating the attributable fraction (AF) and attributable number (AN) at the national level is standard in multi-location DLNM studies. The England-wide pooled exposure–response function you already compute is sufficient to derive a national AF/AN, and I strongly suggest doing so would substantially strengthen the policy relevance of the paper. Policymakers generally rely on AF/AN to quantify burden, as these measures translate relative risks into an interpretable number of additional hospital admissions attributable to heat. If the authors strongly prefer not to include these estimates, I recommend acknowledging this as a limitation and providing a clearer justification, given their central importance for public health planning.*

   Thank you for your comments. As stated in our response to query 3.18, we do agree that attributable fractions (AF) and attributable numbers (AN) are a useful tool to facilitate a broader understanding of heat-related burden particularly for policy makers. Indeed, the authors developed methodologies for deriving attributable risk measured from these types of time series models (Gasparrini & Leone, BMC Med Res Meth 2014), and applied them in large-scale small-area analyses of health effects of temperature-related mortality (Gasparrini et al, Lancet Planetary Health 2022). However, for the same reasons outlined earlier in response to Comment 4, the authors did not consider it appropriate to replicate the same analytical plan for this analysis. An emblematic example is the results for cardiovascular causes, whereby it would be difficult to interpret, and potentially misleading, to provide estimates of ‘avoided’ hospital admissions related to heat, given the negative associated risks for this set of causes. The authors discuss possible explanations for these results in paragraph 3 of the Discussion, in particular pre-hospital mortality. As discussed above, these reasons necessitate the adoption of simpler summary estimates and more elaboration on the results.

7) *Thank you for the response in 3.19 and 3.20. I accept that cost estimation and climate-change projections are beyond the current scope and appreciate the authors identifying these as valuable areas for future work. I suggest briefly noting these potential extensions in the discussion.*

   We acknowledge that this is a valid and important area of further research. This point is now explicitly mentioned in paragraph 4 of the Discussion.

8) *The manuscript states that statistical code is available at the referenced GitHub repository (github.com/gmflower/UK-TRH), but the repository currently contains no code/materials. Please correct the link and/or upload the full analysis code. If the repository is private or not yet public, please make it publicly accessible prior to publication.*

Thank you for highlighting this. The code repository has now been made publicly available. Please note that it is not possible to share the underlying data used in this study, but the code has been made available for illustrative purposes.

VERSION 3 - REVIEW

**Reviewer**	**3**
**Name**	**Varghese, Blesson**
**Affiliation**	**The University of Adelaide Faculty of Health and Medical Sciences**
**Date**	**19-Mar-2026**
**COI**	

Thank you to the authors for their careful revisions and detailed responses to the previous round of comments. The manuscript has improved substantially, particularly with respect to clarification of the APC dataset structure, refinement of adult age group categories, and the addition of future research directions in the discussion. I also appreciate the correction of the code repository access.

However, several important issues remain insufficiently addressed and should be resolved prior to publication.

First, the exclusion of the paediatric population (0–17 years) is now acknowledged, but the justification provided remains largely conceptual rather than methodological. Given the availability of national administrative data and the established literature on paediatric heat vulnerability, a clearer explanation of the analytical or practical rationale for excluding this age group is needed. At minimum, this decision should be explicitly framed as a limitation in the manuscript.

Second, the request for simple overall sex-stratified estimates has not been adequately addressed. The rationale provided, that such analyses were not part of the original plan and would require additional space, is not methodologically compelling, as basic sex stratification is standard practice in environmental epidemiology and would materially strengthen the interpretability and policy relevance of the findings without introducing small-cell concerns. If the authors elect not to include these analyses, a more substantive justification should be provided.

Third, while I acknowledge the authors' rationale for maintaining a national focus on cause-specific comparisons, the absence of any spatial or vulnerability analysis remains a notable omission given the modelling framework and data resolution available. Even if such analyses are beyond the intended scope, this should be explicitly acknowledged as a limitation and

briefly justified, as differential vulnerability is central to climate-health research and public health planning.

Fourth, although the use of LSOA-level temperature assignment represents a methodological strength, residual exposure misclassification is inherent to ecological time-series studies and should be acknowledged explicitly as a limitation, consistent with reporting norms in environmental epidemiology.

Finally, the decision not to estimate attributable fractions or attributable numbers is understandable given the complexities associated with cause-specific interpretations; however, given their central importance for policy translation, this omission should also be clearly acknowledged and justified in the discussion.

In summary, the study is methodologically robust and addresses an important research gap in cause-specific heat-related hospital admissions. However, further clarification and additional transparency regarding scope decisions and limitations are required before I can recommend acceptance.

I therefore recommend minor revision.

VERSION 3 - AUTHOR RESPONSE

Reviewer 3

Thank you to the authors for their careful revisions and detailed responses to the previous round of comments. The manuscript has improved substantially, particularly with respect to clarification of the APC dataset structure, refinement of adult age group categories, and the addition of future research directions in the discussion. I also appreciate the correction of the code repository access.

We thank the Reviewer for their previous comments, which helped improve the manuscript considerably. We note that, in this review, the Reviewer had made very similar comments, reiterating issues that we hoped we had clarified in the two previous rounds. We therefore take the opportunity to provide further clarifications.

Before addressing specific comments, however, we believe it is paramount to point out a couple of aspects that motivated our previous responses.

First, choices regarding the study population or the selected analyses and results were not dictated by ‘methodological’ reasons, but by the framing of the research question. As now clearly defined by its title, this manuscript focuses on the evaluation and comparison of cause-specific risks of hospitalisation in the adult population of England. Additional analyses on a different population (0-17) or by sex and area can be interesting, but they are not within the scope of this research question.

Second, the Reviewer asked to acknowledge as limitations the omission of such additional analyses. However, we consider limitations only those issues that prevent or restrict the ability to address a given research question. In this case, the analysis of the paediatric population, as well as the assessment of sex or area-specific risks, are clearly interesting

topics, but their omission does not limit in any way the ability of our investigation to compare cause-specific risks of hospitalisation in the adult population in England.

We provide more specific comments as responses to the comments below.

However, several important issues remain insufficiently addressed and should be resolved prior to publication.

1) *First, the exclusion of the paediatric population (0–17 years) is now acknowledged, but the justification provided remains largely conceptual rather than methodological. Given the availability of national administrative data and the established literature on paediatric heat vulnerability, a clearer explanation of the analytical or practical rationale for excluding this age group is needed. At minimum, this decision should be explicitly framed as a limitation in the manuscript.*

   As explained in our introductory response above, we provided a detailed explanation about the focus on the adult population in our analysis. Indeed, the exclusion of the 0-17-year-olds is not methodological, but related to the different risk profile for such a heterogeneous age group, which would require separate analyses and interpretations. As mentioned in our response to previous comments, this was acknowledged and actually recommended also by Reviewer 1. In addition, as explained above, we do not consider the omission of results for the 0-17 age group as a limitation in an analysis focused on the adult population. This scope is clearly stated in the title and abstract of the manuscript.

2) *Second, the request for simple overall sex-stratified estimates has not been adequately addressed. The rationale provided, that such analyses were not part of the original plan and would require additional space, is not methodologically compelling, as basic sex stratification is standard practice in environmental epidemiology and would materially strengthen the interpretability and policy relevance of the findings without introducing small-cell concerns. If the authors elect not to include these analyses, a more substantive justification should be provided.*

   We have given your comments careful further consideration, however, we respectfully maintain our position that sex-stratified analyses fall outside the scope of the current manuscript.

   First, we respectfully disagree that basic sex stratification is standard practice in environmental epidemiology. It is quite the opposite, as only a few of the major studies on the health effects of temperature report sex-specific risks. For instance, a recent systematic review on heat exposure and cardiovascular health outcomes co-authored by the Reviewer (https://pubmed.ncbi.nlm.nih.gov/35709806/) shows how a small minority of peer-reviewed analyses, specifically 13.2% (20 out of 151) for mortality and 24.6% (30 out of 122) for morbidity, reported sex-specific risks,

   More importantly, the authors disagree with the expectation that a simple overall sex-stratified estimate would be sufficient here. We believe that presenting stratified

estimates without adequately interrogating the underlying mechanisms — including potential confounding by sex-specific age structures — risks generating findings that are potentially misleading to readers and policymakers. The analysis the reviewer envisions as straightforward would, in our view, demand a depth of treatment that is not feasible within the constraints of the present paper.

Finally, the current manuscript was designed with specific research questions in mind, and the addition of sex-stratified analyses would represent a meaningful expansion of scope rather than a minor supplementary step. We therefore maintain that our original justification — that these analyses were not pre-specified and exceed the intended scope of this work — remains both methodologically and editorially valid. We would welcome the opportunity to address sex-related differences as a primary focus in a dedicated future analysis, where it can be given the rigour it deserves.

3) *Third, while I acknowledge the authors' rationale for maintaining a national focus on cause-specific comparisons, the absence of any spatial or vulnerability analysis remains a notable omission given the modelling framework and data resolution available. Even if such analyses are beyond the intended scope, this should be explicitly acknowledged as a limitation and briefly justified, as differential vulnerability is central to climate-health research and public health planning.*

   Thank you for your comments. The authors agree that this is a limitation of this analysis, stemming from highly volatile local risks. This point is acknowledged in paragraph 4 of the Discussion section, stating: "*More importantly, this study reports country-wide estimates and did not attempt to identify local differences in risks within England*".

4) *Fourth, although the use of LSOA-level temperature assignment represents a methodological strength, residual exposure misclassification is inherent to ecological time-series studies and should be acknowledged explicitly as a limitation, consistent with reporting norms in environmental epidemiology.*

   Thank you for your comments. The authors have amended the Discussion section of the manuscript accordingly. The relevant text (Discussion section, fourth paragraph) is included below for reference:

   "Although this study benefits from high resolution mapping of temperature exposure, there remains a minor risk of exposure misclassification, especially in the more rural LSOAs".

5) *Finally, the decision not to estimate attributable fractions or attributable numbers is understandable given the complexities associated with cause-specific interpretations; however, given their central importance for policy translation, this omission should also be clearly acknowledged and justified in the discussion.*

   Thank you for acknowledging the difficulty in representing heat-related burden as attributable numbers or fractions, particularly in the case of the cardiovascular results

provided in this study. The authors have carefully considered this repeated suggestion, however, for the reasons clearly outlined in our previous response, we have decided not to acknowledge this as a limitation of this study. Rather, we see it as an appropriate choice not to include these measures, as doing so could lead to misleading interpretations. We believe that the reporting of the health risk in terms of the effect summaries selected in our analysis provides a balanced overview and satisfactorily addresses the research question.

In summary, the study is methodologically robust and addresses an important research gap in cause-specific heat-related hospital admissions. However, further clarification and additional transparency regarding scope decisions and limitations are required before I can recommend acceptance.
